# Innovative Technologies Reshaping Meat Industrialization: Challenges and Opportunities in the Intelligent Era

**DOI:** 10.3390/foods14132230

**Published:** 2025-06-24

**Authors:** Qing Sun, Yanan Yuan, Baoguo Xu, Shipeng Gao, Xiaodong Zhai, Feiyue Xu, Jiyong Shi

**Affiliations:** 1 School of Food and Biological Engineering, Jiangsu University, Zhenjiang 212013, China; 2 Guangdong Provincial Key Laboratory of Intelligent Food Manufacturing, Foshan University, Foshan 528225, China

**Keywords:** artificial intelligence, meat industrialization, smart cutting, non-thermal processing, blockchain traceability

## Abstract

The Fourth Industrial Revolution and artificial intelligence (AI) technology are driving the transformation of the meat industry from mechanization and automation to intelligence and digitization. This paper provides a systematic review of key technological innovations in this field, including physical technologies (such as smart cutting precision improved to the millimeter level, pulse electric field sterilization efficiency exceeding 90%, ultrasonic-assisted marinating time reduced by 12 h, and ultra-high-pressure processing extending shelf life) and digital technologies (IoT real-time monitoring, blockchain-enhanced traceability transparency, and AI-optimized production decision-making). Additionally, it explores the potential of alternative meat production technologies (cell-cultured meat and 3D bioprinting) to disrupt traditional models. In application scenarios such as central kitchen efficiency improvements (e.g., food companies leveraging the “S2B2C” model to apply AI agents, supply chain management, and intelligent control systems, resulting in a 26.98% increase in overall profits), end-to-end temperature control in cold chain logistics (e.g., using multi-array sensors for real-time monitoring of meat spoilage), intelligent freshness recognition of products (based on deep learning or sensors), and personalized customization (e.g., 3D-printed customized nutritional meat products), these technologies have significantly improved production efficiency, product quality, and safety. However, large-scale application still faces key challenges, including high costs (such as the high investment in cell-cultured meat bioreactors), lack of standardization (such as the absence of unified standards for non-thermal technology parameters), and consumer acceptance (surveys indicate that approximately 41% of consumers are concerned about contracting illnesses from consuming cultured meat, and only 25% are willing to try it). These challenges constrain the economic viability and market promotion of the aforementioned technologies. Future efforts should focus on collaborative innovation to establish a truly intelligent and sustainable meat production system.

## 1. Introduction

Food industrialization refers to the transformation of food production from traditional manual processes to large-scale, high-efficiency systems through mechanization, standardization, and automation. The concept began to take shape in the 18th century, spurred by wartime demands, with technological milestones such as Nicolas Appert’s invention of canning and Louis Pasteur’s development of pasteurization laying the foundation for modern food preservation and safety systems [1,2]. The Industrial Revolution further accelerated this transformation by introducing machinery into food preparation, production, and distribution, leading to the rise of large-scale mechanized operations [3].

Meat, as a vital component of the food sector, has undergone a parallel trajectory of industrial transformation. In 1870, the Cincinnati Meat Works introduced the first meat assembly line, marking a significant step toward the mechanization of meat processing [4]. However, the frequent occurrence of foodborne illnesses caused by pathogens such as *E. coli* and *Listeria* in the 20th century sparked public concern about meat safety and quality [5,6]. This prompted the establishment of robust regulatory systems, exemplified by the EU’s adoption of the Hazard Analysis and Critical Control Points (HACCP) framework in the 1990s [7]. As a result, modern quality assurance protocols became integral to meat processing. By the late 20th century, automation had been widely adopted in primary processing steps such as slaughtering, defeathering, and segmentation. These advancements significantly increased throughput—from 3000 to over 10,000 units per hour—and enhanced hygiene through improved cold chain logistics and safety standards [4,8].

In recent years, the deep integration of information technology, computer science, and advanced engineering has been driving a new round of transformation in the meat industry, characterized by the rapid adoption of intelligent and digital solutions [9]. Disruptive technologies such as artificial intelligence (AI), the Internet of Things (IoT), digital twins (DT), and robotics are reshaping industry operational models [10,11]. In the field of processing technology, non-thermal processing techniques such as high-pressure processing (HPP) and pulsed electric field (PEF) are emerging as alternatives to traditional thermal processing, demonstrating advantages in improving protein functionality, efficiently inactivating microorganisms, and extending product shelf life [12]. For example, by precisely controlling pressure parameters, HPP technology effectively enhances the protein functional characteristics of cured meat while achieving the dual objectives of microbial inactivation and extended shelf life [13]. In terms of intelligent equipment, three-dimensional modeling systems based on machine vision can guide robotic arms to achieve high-precision cutting, while multi-modal sensing technologies such as X-ray and ultrasound can achieve millimeter-level cutting precision [4]. In quality monitoring, hyperspectral imaging technology utilizes spectral information within the 400–500 nm range to enable the real-time, non-destructive detection of pork quality characteristics such as pale color, soft texture, and exudation [13].

**Figure 1:**
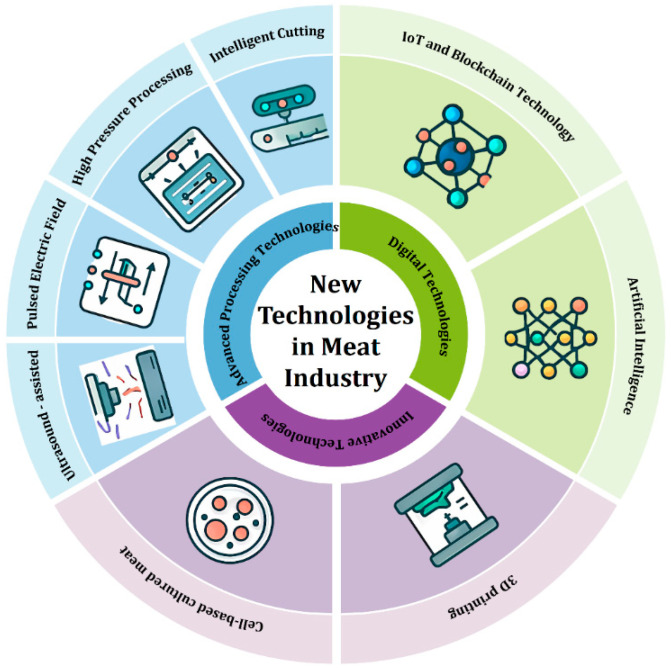
New technologies in the meat industry.

The meat industry is currently undergoing a profound paradigm shift driven by the Fourth Industrial Revolution, with its core characteristic being the transition from automation to intelligence and digitization. Unlike previous reviews, the unique contribution of this review lies in its systematic integration and analysis of the two innovative forces reshaping the meat industry: physical technological innovation and digital intelligent systems (as illustrated in Figure 1). The aim is to bridge the gap between traditional food engineering and cutting-edge information science, revealing the synergistic effects of their integration, thereby providing a comprehensive framework for constructing the next-generation intelligent meat production system.

To systematically elucidate this transition from “automated factories” to “intelligent ecosystems,” this paper will focus on the following core modules: First, it reviews the physical processing technologies directly applied to meat products. These include non-thermal processing technologies that enhance quality and safety (such as HPP and PEF), as well as intelligent equipment that improves efficiency and precision (such as machine vision-based precision cutting robots and multi-modal sensing systems). Second, it analyzes how digital technologies can be used to optimize the entire production process. This includes the application of the IoT in real-time monitoring, the role of blockchain in enhancing supply chain transparency and traceability, and the core value of AI in production decision-making, quality prediction, and energy consumption management. Next, it explores alternative technologies beyond traditional frameworks, focusing on cutting-edge technologies such as cell-cultured meat and 3D bioprinting, and assesses their potential to disrupt the existing meat supply chain, as well as the scientific, ethical, and market challenges they face. Finally, the paper describes the comprehensive application of these technologies in real-world scenarios such as central kitchens, cold chain logistics, and personalized customization, and delves into the key obstacles hindering their widespread deployment, including high initial investment costs, the absence of industry standards, data security risks, and consumer acceptance, while conducting a cautious analysis of their economic feasibility.

Through this structured analysis, this paper aims to provide academia and industry with a comprehensive overview of the intelligent transformation of the meat industry and to guide future research and investment directions.

## 2. Technological Innovation Systems

### 2.1. Advanced Processing Innovations

#### 2.1.1. Intelligent Cutting Technology

Cutting is a fundamental process in meat processing, transforming raw materials into specific shapes and sizes to facilitate downstream operations, enhance yield, and meet diverse product demands. Traditional cutting methods rely heavily on manual labor [14], which presents multiple challenges, including harsh working conditions [15], high physical and mental strain on workers [16], difficulties in workforce recruitment [17], and rising labor costs. These factors hinder sustainable development. The emergence of intelligent cutting systems offers a promising solution to address these limitations.

Intelligent cutting integrates AI, machine vision, and robotics to automate meat processing tasks with high precision. Equipped with 3D sensors and real-time recognition capabilities, these systems detect meat characteristics, plan optimal cutting paths using AI algorithms, and control robotic arms to execute fine and consistent segmentation with minimal human intervention. This technological shift addresses the variability in meat tissue, structure, and dimensions, which demand exceptional adaptability and precision.

At the core of intelligent cutting systems is the robotic cutting unit, where the robotic arm serves as the critical hardware component. Due to the heterogeneous nature of meat—including variability in type, structure, tissue composition, and size—cutting operations demand exceptional accuracy. Multiple factors must be considered, including dimensional specifications, tissue distribution, cutting trajectories, tool positioning, and actuation force parameters. Recent advancements illustrate the importance of integrating sensing and intelligent algorithms to enhance performance. For instance, RGB-D cameras have been used to capture spatial data from different pork cutting positions, improving object recognition and segmentation accuracy in robotic systems [18]. the inverse consistent deep neural network (ICNet) deep learning model enables precise anatomical localization in sheep carcass segmentation, achieving 97.68% accuracy [19]. Time-delay neural networks (TDNNs) allow real-time trajectory adjustment with success rates between 86% and 90% by integrating force and position signals [20]. In another example, Mason developed a smart cutting tool based on electromagnetic induction that achieved a contact detection error as low as 1.78% [21]. Table 1 summarizes additional case studies that highlight the synergistic application of algorithmic modeling and sensor fusion in intelligent meat cutting.

In fully automated meat processing environments, hygiene and safety management are critical to maintaining stable and efficient operations [16]. Processing facilities face multiple risks such as microbial contamination, foreign object intrusion, and corrosion due to high humidity levels [22]. To mitigate these risks, several strategies have been proposed: (i) surface treatment of equipment using antimicrobial materials such as silver nanocoatings and photocatalytic layers to inhibit biofilm formation [23]; (ii) deployment of automated cleaning procedures and clean-in-place (CIP) systems, with real-time monitoring of cleaning agent concentrations using conductivity sensors to ensure sterilization effectiveness [24]; and (iii) installation of anti-splash mechanisms and vacuum adsorption devices to reduce aerosol dispersion and airborne microbial contamination [25,26]. Additionally, emergency braking systems equipped with multimodal sensors (e.g., pressure, temperature, and vision) can detect anomalies and trigger millisecond-level responses to prevent accidents.

Despite demonstrating significant potential, the actual deployment of intelligent cutting systems still requires consideration of multiple factors. Existing research indicates that the system performs exceptionally well in terms of identification and localization accuracy (e.g., using a multispectral imaging system to detect bone fragments in pork slices with an accuracy rate of 90% [27]) and specific path planning (e.g., Zhang et al. proposed a method based on deep reinforcement learning (DRL) that can automatically generate rule-compliant and efficient process routes [28]). However, the overall processing speed still lags behind that of highly skilled workers, particularly when handling highly irregular carcasses [16,29]. To transition from laboratory research to large-scale industrial application, current intelligent cutting technology must overcome numerous challenges, including high costs, compatible and safe cutting systems, material durability and regular maintenance, and compatibility with different meat types [30]. Continuous technological innovation and iterative optimization are required, along with enhanced interdisciplinary collaboration to improve the integration and reliability of the system, enabling it to play a more significant role in future intelligent manufacturing.

Driven by Industry 4.0, cutting systems are increasingly integrated with digital technologies—such as IoT, big data analytics, blockchain, and digital twins—enabling enhanced traceability, precision, and coordination along the value chain [10,31,32,33]. Their application facilitates the optimization of human–machine collaboration, enhances processing accuracy and efficiency [34], and enables a seamless transition from slaughtering to intelligent segmentation, real-time quality monitoring, and cold-chain logistics [35]. For example, blockchain-based traceability systems can ensure data transparency and immutability, thereby establishing trustworthy food safety mechanisms and enhancing consumer confidence in meat products processed through intelligent systems [29].

**Table 1:** Sensors combined with machine algorithms for intelligent cutting of meat.

References	Research Purpose	Research Methodology	Main Findings
[18]	Providing pig carcass cutting datasets to help develop intelligent systems for the meat industry	Acquisition of RGB-D data from 25 pigs with 6 cameras, stored as bag files and paired with JSON files	The dataset contains a variety of parameters, which can be used for multi-disciplinary research and promote the development of relevant robots and intelligent systems
[19]	Realizing real-time semantic segmentation of sheep carcass images	Acquisition, enhancement of sheep carcass images, labeling to construct the dataset, experimentation with ICNet models	ICNet model segmentation with high accuracy, good real-time performance, and good generalization ability
[20]	Enabling robots to recognize contact states for improved operational flexibility	Cutting with a robotic arm, TDNN recognizes objects and plans movement trajectories	TDNN can distinguish object characteristics and motion planning improves processing capability, but recognition accuracy needs to be improved
[21]	Developing smart knives with real-time feedback for robotic meat cutting	Design of smart tools, utilizing EM wave sensing technology, validated by simulation and experimentation	The tool can determine contact and depth more accurately, providing intelligent feedback for robotic cutting, with room for improvement
[36]	Development of localization and cutting point algorithms for trout processing systems	Trout images were acquired and cut points were extracted by preprocessing and segmentation steps	Algorithms accurately detect relevant parts and fins to determine the cutting point and automate the process
[37]	Exploring the use of haptic sensing in robotic red meat cutting	Cutting slices of beef tenderloin with a knife with a force sensor and analyzing the force signal	Force signals are recognizable, and tactile perception distinguishes between tissue and cutting phases, providing the basis for control strategies
[38]	Realization of automatic identification and classification of pork cut parts	Images are collected, preprocessed, and recognized based on a modified ResNet-50 model	The method has a recognition accuracy of 94.47% but is affected by the dataset and image environment
[39]	Development of a pHRI-based meat-cutting assistance strategy to reduce personnel illnesses	Develop an impedance control system, design two strategies and experiment with them	Determination of suitable sensors, advantages of both strategies, comfortable system operation

#### 2.1.2. Pulsed Electric Field Technology

PEF is an emerging non-thermal food processing technology that utilizes short bursts of high-intensity electric fields—typically ranging from 0.1 kV/cm to 80 kV/cm—for the treatment of food materials [40,41]. The core mechanism underlying PEF is electroporation [42], wherein the electric field induces transient or permanent structural rearrangements in the phospholipid bilayer of cell membranes, thereby significantly enhancing membrane permeability [43]. Compared to conventional thermal processing methods, PEF offers several advantages, including minimal heat generation, ultra-short treatment duration, and compatibility with continuous production systems [44]. Furthermore, PEF has been shown to affect protein secondary and tertiary structures by modulating intermolecular interactions (e.g., hydrogen bonding, hydrophobic interactions), thus influencing protein functionality [45].

Although PEF technology has been extensively explored for microbial inactivation in liquid food systems—such as milk, fermented dairy, fruit juices, beer, and alcoholic beverages [46,47,48,49,50,51]—its application in meat processing has gained increasing attention, particularly in marination and thawing processes, due to its unique membrane-disruptive effects.

During marination, the electroporation effect induced by PEF compromises cell membrane integrity, greatly facilitating the diffusion and penetration of marinade constituents (e.g., salts, nitrates/nitrites, sugars, spices, and flavor compounds) into meat tissues, including intracellular spaces [41]. This enhanced mass transfer not only reduces the marination time and increases process efficiency, but it also enables the development of low-sodium or “healthier” cured products by achieving desired flavor and preservation outcomes at reduced additive concentrations. Additionally, PEF contributes to more uniform solute distribution within the tissue matrix, thereby improving flavor homogeneity and overall product quality.

In the context of thawing frozen meat, PEF has demonstrated the ability to modify the microstructure and dynamics of ice crystals and their surrounding environments. The electric field may activate water molecules at the ice–water interface, accelerating the melting process. It may also induce the recrystallization or morphological alteration of ice crystals, thereby enhancing thawing uniformity [52]. More importantly, PEF-induced increases in membrane permeability enhance water redistribution and mass transfer during thawing, facilitating quicker osmotic balance across cell membranes. This leads to reduced drip loss, improved moisture retention, and more efficient heat transfer to the meat’s interior, thereby shortening the total thawing time [53].

The efficacy of PEF in meat processing is highly dependent on the optimization of key parameters such as electric field strength, pulse duration, frequency, treatment time, and temperature. Low-intensity fields (<1 kV/cm) typically result in reversible membrane permeability, which is beneficial for marination and thawing, while high-intensity fields (>20 kV/cm) can cause irreversible membrane rupture and microbial inactivation. However, excessive intensity or duration can negatively impact meat quality by damaging muscle fiber microstructures, potentially leading to reduced tenderness, water-holding capacity, and undesirable color changes [54].

Recent studies have provided empirical evidence for these effects. Guo et al. reported that PEF treatment accelerated marination by 66.7%, significantly improving moisture content and textural properties in beef [55]. Li et al. demonstrated that low-intensity PEF reduced thawing time by 20 min, decreased total drip loss by 6%, and enhanced the structural integrity and freshness of muscle fibers [56]. In another study, Karki et al. found that electric field intensity and sample conductivity directly influenced meat quality and color in bone-in beef ribs, offering a framework for parameter optimization in complex meat systems [57]. Table 2 summarizes selected studies on PEF-assisted meat marination and thawing, highlighting parameter settings and observed outcomes.

In conclusion, PEF presents a promising technological pathway for improving the efficiency, quality, and nutritional profile of processed meat products. However, its successful industrial application requires the precise tailoring of process conditions based on meat type, anatomical location, and processing goals (e.g., marination, thawing, tenderization), to maximize benefits while minimizing potential adverse effects.

In addition, its large-scale industrial application still faces some key challenges. A major bottleneck of PEF systems is their high energy requirements [58]. PEF processing requires the generation of high-voltage pulses, which inevitably leads to energy consumption. To reduce energy requirements, the design and operating parameters of PEF systems, such as pulse waveform, frequency, and electric field strength, can be optimized to reduce energy waste [59].

The irregular geometry of meat poses challenges for PEF processing in terms of uniform electric field distribution [58]. If the electric field strength is uneven, it will affect the processing effectiveness and further impact the quality of meat products. To address this issue, the shape of meat blocks can be reshaped to improve electric field distribution; electrode design can be optimized by designing appropriate electrode shapes and arrangements to achieve more uniform electric field distribution [60].

For products with bones or high fat content, processing effectiveness is often limited [58]. The electrical conductivity of bones and fat differs from that of muscle, leading to distorted electric field distribution and reduced PEF processing effectiveness. Adjusting electric field parameters, optimizing field strength, pulse width, and frequency [57]; removing some bones or fat during pre-processing to enhance PEF processing effectiveness; and combining PEF with other technologies to achieve synergistic effects are all viable approaches. For example, combining PEF with ultrasound improves chicken breast quality and enhances processing effectiveness [61].

Through further research and technological innovation, the advantages of PEF technology can be fully leveraged to provide consumers with higher-quality, safer meat products.

**Table 2:** Application of pulsed electric field technology to cured and defrosted meats.

References	Research Purpose	Research Methodology	Main Findings
[55]	Study of the effect of PEF parameters on beef curing and quality	Beef was marinated after treatment with different field strengths (2–4 kV/cm) and time (60–90 s) to analyze NaCl diffusion, moisture, texture, and structure	PEF accelerated NaCl penetration and shortened curing time by 66.7%, and the 4 kV/cm + 60 s treatment enhanced quality best
[56]	Investigating the role of PEF on the quality of freeze-thawed Atlantic salmon	PEF treatment of frozen–thawed salmon to analyze thawing time, fiber structure, and water holding capacity	PEF reduces thawing time by 20 min, reduces losses by 6%, and improves texture but slightly increases oxidation
[57]	Exploring the association between the conductivity of bovine calves and the effect of PEF treatment	Beef chops with different conductivities were analyzed for texture and color after PEF treatment combined with low-temperature slow cooking.	PEF improves tenderness, conductivity affects required processing time, and PEF reduces processing variability
[62]	Analyzing the effect of PEF on the quality and metabolites of wet/dry cured venison meat	High-and low-intensity PEF treatment of venison for tenderness, metabolites, and drying rates	High-intensity PEF increased tenderness by 9% and drying rate by 6%, with metabolite differences mainly caused by curing method
[63]	Evaluating the effect of PEF on the quality of fresh beef and frozen–thawed meat	PEF treatment of fresh and frozen–thawed beef for determination of color, oxidation, and sensory parameters	PEF improves tenderness and color but increases oxidation of freeze-thawed meat and affects sensory sensation when stored for 7 days
[64]	Exploring the effect of PEF-assisted thawing on duck meat quality	Determination of thawing rate, protein structure, and texture of duck meat thawed by PEF with different field strengths	1–3 kV/cm PEF reduces thawing time by 50%, reduces losses and maintains meat quality, and stabilizes water holding capacity
[65]	Examining the effect of PEF pretreatment on pork curing efficiency	Needle electrode PEF treatment of pork after pickling system to analyze NaCl penetration, proteins, and microstructure	3 kV/cm PEF shortens curing time 12 h and promotes salt penetration by widening the muscle gap

#### 2.1.3. Ultrasound-Assisted Processing Technology

As a green and environmentally friendly non-thermal processing method, ultrasound technology has gained significant attention in the field of food science and engineering due to its wide range of applications [66,67]. The functional outcome of ultrasonic treatment largely depends on the frequency and intensity of the ultrasound employed. High-frequency, low-intensity ultrasound (typically 100 kHz to 1 MHz, <1 W/cm^2^) is primarily used for non-destructive testing of food products [68,69], enabling real-time monitoring of key physicochemical parameters such as sugar content, hardness, and ripeness [70]. In contrast, ultrasound with frequencies ≥20 kHz is commonly applied in food processing operations [71].

When applied to protein-rich food systems such as meat, ultrasound induces a series of physicochemical changes [72,73,74], with acoustic cavitation serving as the primary mechanism. The collapse of cavitation bubbles generates localized high temperatures and pressures, intense shear forces, and micro-jets, which can lead to conformational alterations in proteins and even partial peptide bond cleavage [75]. The efficiency of ultrasound treatment is governed by multiple interacting parameters, including frequency, power, intensity, duration, and system temperature [76,77]. Fine-tuning these variables is crucial to achieving optimal processing outcomes while minimizing quality degradation.

Following slaughter, skeletal muscle undergoes complex postmortem biochemical transformations. Rapid ATP depletion and anaerobic glycolysis lead to lactic acid accumulation and a gradual pH decline toward the isoelectric point, causing muscle stiffening (rigor mortis) [78]. If unmanaged, this process may result in decreased tenderness and water-holding capacity. Ultrasound offers a promising physical intervention, as its mechanical vibration and cavitation effects can disrupt myofibrillar integrity, relax inter-fiber linkages, and activate endogenous proteases. These mechanisms collectively enhance meat tenderness, improve water retention, and preserve juiciness.

Curing is a pivotal process in meat preservation and flavor enhancement, typically involving the diffusion of curing agents such as salts, sugars, and spices into muscle tissue [79]. Traditional curing methods (e.g., dry or wet curing) rely on passive diffusion, which is inherently slow and prone to uneven solute distribution. The simultaneous outward migration of water and inward diffusion of curing agents constitutes a complex mass transfer process, influenced by factors such as brine concentration, temperature, and tissue structure (e.g., fiber density, fat content, and distribution) [80,81].

Ultrasound-assisted curing provides an effective strategy to enhance curing efficiency. Several studies have demonstrated that ultrasonic treatment can significantly accelerate curing while maintaining product quality [82]. For instance, Lin et al. reported that 90 min of ultrasound followed by 12 h of static curing achieved results comparable to 24 h of conventional curing [83]. Inguglia et al. found that reducing the distance between the ultrasound probe and the meat sample to 0.3 cm maximized NaCl diffusion rates [84]. Guo et al. demonstrated that ultrasound at 26.8 kHz enhanced water-holding capacity by promoting free water conversion and accelerating salt diffusion [85]. The underlying mechanism is attributed to ultrasound-induced disruption of cell membranes and increased tissue porosity, which creates additional pathways for marinade penetration [80]. This facilitates faster and more uniform distribution of curing agents, improving the flavor intensity, texture, and overall sensory attributes of the final product [86].

Freezing and thawing rates are critical determinants of meat quality. Slow freezing or thawing promotes the formation of large ice crystals, which can physically damage cellular structures and result in excessive drip loss, reduced tenderness, and nutrient depletion upon thawing [87,88]. Ultrasound has been found to positively modulate both processes. During freezing, ultrasound-induced cavitation generates numerous nucleation sites, promoting the rapid formation of fine ice crystals and shortening freezing time, thereby mitigating structural damage [89,90]. During thawing, ultrasound enhances heat and mass transfer through cavitation, microstreaming, and moderate thermal effects, accelerating ice crystal melting and ensuring uniform thawing. This avoids surface overheating and internal under-thawing, preserving the structural integrity and quality of the meat [91].

Several studies have substantiated the benefits of ultrasound-assisted thawing. For example, single-frequency ultrasound reduced thawing times of goose meat by 45.37–57.58% [92], while multi-frequency ultrasound shortened beef thawing time by 15.7–45.4% compared to water thawing [93]. In another study, 20 kHz ultrasound at varying power densities (0–40 W/L) significantly reduced pork thawing time, with optimal results at 40 W/L [94]. Dual-frequency ultrasound also reduced goose thawing time by 17.76–36.06% [95]. Furthermore, thawing losses in pork, beef, and lamb were reduced by approximately 43%, 45%, and 43%, respectively, compared with conventional thawing methods [96].

Although ultrasonic technology has demonstrated significant advantages in meat processing, its industrial application still faces the following challenges. The cavitation effects and free radicals generated by ultrasonic waves may cause lipid oxidation in meat rich in unsaturated fatty acids (such as poultry and fish), affecting flavor stability and shelf life [97]. Therefore, during ultrasonic processing, it is essential to control and optimize processing parameters (such as reducing temperature and shortening processing time) or incorporate antioxidants to minimize lipid oxidation [98]. Additionally, ultrasonic waves have limited penetration capabilities, and for large pieces of meat or irregularly shaped samples, ultrasonic energy may not distribute uniformly, resulting in inconsistent processing effects between the interior and exterior of the meat product [99]. For uneven processing, techniques such as multi-frequency or pulsed ultrasound can be employed to improve the energy transmission medium and enhance energy penetration and uniformity [95].

When applying ultrasonic technology to the meat industry, factors such as energy transmission efficiency, equipment durability, and cost–benefit analysis must be considered to ensure compatibility with industrial processes [100]. However, certain ultrasonic processing conditions may have adverse effects, such as excessive ultrasonic processing leading to excessive moisture loss in meat products, which can affect the texture and taste of the final product [101]. Therefore, it is also necessary to optimize ultrasonic processing parameters to achieve good compatibility with other processes.

To overcome the limitations of ultrasonic technology, researchers have explored the combined application of ultrasonic waves with other technologies. For example, combining ultrasonic waves with HPP can make meat tissue structures more porous, enhancing ultrasonic penetration and tenderization effects [102]. HPP can also inhibit lipid oxidation caused by ultrasonic waves, extending the shelf life of meat products [98]. Additionally, studies have shown that when ultrasonic waves are combined with PEF, PEF disrupts cell membranes, promoting ultrasonic wave penetration and thereby improving extraction efficiency [103].

Beyond tenderization, marination, freezing, and thawing, ultrasound technology has found broader applications across the meat and food processing industries [104,105,106,107,108]. These include enhancing drying efficiency [109,110,111], improving microbial safety through ultrasonic-assisted sterilization, facilitating enzymatic reactions [112,113,114], and supporting the cleaning of raw materials and processing equipment [115]. Due to its high efficiency, low environmental impact, and compatibility with other processing technologies, ultrasound offers valuable technical support for improving meat product quality, developing novel formulations, and optimizing conventional processing operations.

#### 2.1.4. High-Pressure Treatment Technology

High pressure processing (HPP) is an advanced non-thermal food preservation technology that has been extensively applied across the food industry, accounting for approximately 25–30% of the meat processing sector [116]. By subjecting food products to ultra-high hydrostatic pressures ranging from 100 MPa to 800 MPa, HPP induces structural and functional alterations in microbial cell membranes as well as conformational denaturation of macromolecules such as proteins [117]. These physicochemical changes result in the effective inactivation or elimination of microorganisms, thereby extending product shelf life and enhancing food safety [118].

In meat products, HPP offers benefits beyond microbial inactivation; it also positively influences meat quality attributes such as tenderness, texture, and flavor by modulating protein structures, enzymatic activities, and lipid oxidation pathways [119]. For instance, HPP has been shown to induce partial denaturation or modification of proteins, activate or deactivate specific enzyme systems, and alter the rate of lipid oxidation [120]. These combined effects contribute to enhanced palatability, particularly by improving tenderness and juiciness [121].

HPP has demonstrated significant advantages in meat curing, freezing, and thawing by addressing key limitations of conventional methods. Traditional marination processes are often time-consuming and result in non-uniform diffusion of curing agents, compromising product quality. High-pressure treatment accelerates mass transfer by altering the muscle tissue structure, enabling faster and more homogeneous penetration of salts and flavor compounds [122]. This enhancement significantly shortens marination time while improving its efficiency and consistency. Moreover, HPP enhances protein–water interactions, increases water-holding capacity, reduces juice loss, and improves both yield and sensory quality of the final product [123].

In the context of freezing and thawing, HPP has been applied innovatively to improve product quality. During pressure-assisted freezing, high pressure modifies the freezing point of water and alters the kinetics of ice crystal formation. This promotes the generation of smaller and more uniform ice crystals, which mitigates mechanical damage to muscle cell structures [124]. As a result, thawed meat better retains its microstructure, with reduced drip loss and improved textural integrity and juiciness [125]. For pressure-assisted thawing, HPP enhances thermal conductivity, enabling faster and more uniform thawing [126]. This reduces thawing time and simultaneously inhibits microbial growth and physicochemical deterioration, thereby preserving the structural and sensory properties of the meat.

Several empirical studies support the efficacy of HPP in improving meat quality and extending shelf life. In low-sodium, naturally cured Vienna sausages, treatment at 600 MPa for 3 min extended the shelf life to 12 weeks [127]. A 400 MPa treatment of pork tenderloin improved perceived freshness and increased sensory scores by 20%, primarily through activation of AMP deaminase (AMPD), which facilitates the accumulation of inosine monophosphate (IMP) [128]. In cured hams, 600 MPa treatment offset the adverse textural effects caused by reduced salinity, increased water retention, and lowered shear force by 15–20% [129]. Pressure-assisted thawing at 140 MPa yielded the lowest thawing losses, with elevated pressure promoting protein unfolding and influencing meat quality parameters [130]. In frozen chicken, treatment at 800 MPa for 6 min reduced the total microbial load to below 10^2^ CFU/g while maintaining pH stability in the 6.0–6.2 range, consistent with European Union food safety standards [131].

Overall, HPP holds considerable potential for enhancing the uniformity and efficiency of meat marination, freezing, and thawing processes. By modifying physical structure and optimizing mass transfer, HPP not only improves product consistency and microbiological safety but also helps maintain sensory quality and nutritional value.

However, its widespread adoption still faces the following challenges: The initial equipment investment costs are relatively high, posing a significant economic burden for small and medium-sized processing enterprises. Reducing the cost of HPP equipment or offering more flexible leasing and installment payment options could facilitate the adoption of HPP technology in the meat industry [132]; to ensure that pressure is distributed uniformly throughout the food, HPP technology typically requires food to be packaged in flexible vacuum packaging. Developing lower-cost, high-performance packaging materials is critical for the application of HPP technology in the meat industry [133]; HPP technology may also have certain effects on the sensory attributes of products, such as color, aroma, and texture [134]. Research indicates that HPP can cause changes in the color of meat products and may also affect product tenderness and hardness [135]. Therefore, optimizing HPP process parameters to minimize adverse effects on product sensory qualities can enhance consumer acceptance and promote the application of HPP technology in the meat industry [128].

In the future, HPP technology can be combined with other processing technologies and innovatively integrated into hybrid processing systems (e.g., in conjunction with mild thermal processing [136] or vacuum/modified atmosphere packaging technologies [137]), to achieve complementary advantages, developing more efficient and cost-effective hybrid processing systems that bring greater benefits to the meat industry. Fully leveraging its non-thermal sterilization advantages could reduce reliance on chemical additives such as preservatives [138], and combining it with other natural preservation technologies could drive the development of cleaner-label, healthier meat products [139].

**Figure 2:**
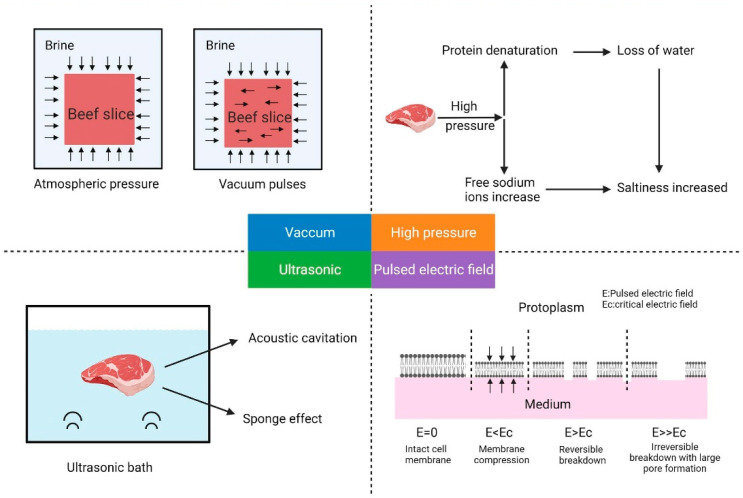
Mechanism of action of process technology marinades [154].

Complementary technologies, including PEF and ultrasound, offer additional pathways for quality enhancement. PEF disrupts cell membranes by delivering brief, high-intensity electric pulses; ultrasonic processing employs high-frequency acoustic waves to induce cavitation, altering meat’s physical structure; and HPP utilizes hydrostatic pressure to disrupt microbial cells and enhance solute diffusion during marination. The specific mechanisms of action for each technology are illustrated in Figure 2. Collectively, these emerging technologies demonstrate promising capabilities in improving meat tenderness, texture, and overall quality. Table 3 further presents the comparative effects of ultrasound and HPP treatments on meat tenderness. Each technology has distinct characteristics, and in some applications, combined use may produce synergistic benefits, resulting in superior processing outcomes and product performance.

#### 2.1.5. Regulatory Landscape and Challenges for Non-Thermal Technologies in Meat Applications

The commercial application of non-thermal processing technologies in the meat industry is not only dependent on their technical effectiveness but is also strictly regulated by health and technical regulations in various countries. Currently, there are significant differences in the regulatory maturity of different technologies, resulting in a landscape ranging from widely accepted to exploratory validation.

HPP is currently the most well-regulated and widely applied non-thermal technology. Although the Codex Alimentarius Commission has not established specific standards for HPP, its application must comply with foundational guidelines such as the General Principles of Food Hygiene. The U.S. Food and Drug Administration (FDA) and the U.S. Department of Agriculture Food Safety and Inspection Service (USDA-FSIS) have explicitly recognized HPP as an effective sterilization technology, focusing primarily on its impact on food safety and establishing corresponding regulations [140]. HPP technology is regarded as an effective food preservation method in the European Union, capable of replacing traditional heat treatment. It is widely used to extend product shelf life and is considered one of the industry standard practices, regulated by general food safety regulations such as Regulation (EC) No 178/2002 [141]. China maintains an open attitude toward the application of HPP technology. Although there are no specific national standards targeting it, its application must comply with the overall requirements of the Food Safety Law and ensure that the final product meets national standards [140].

In contrast, the regulatory pathways for PEF and ultrasonic-assisted technologies are more complex. PEF technology has gained partial recognition for its application in liquid foods such as juices, but its use in solid foods like meat remains in the developmental stage. PEF for meat processing is likely to fall under the scope of the Novel Food Regulation (Regulation (EU) 2015/2283) [142]. This means that before it can be applied commercially, an application must be submitted and undergo a rigorous safety assessment. The FDA requires that PEF technology used for sterilization or processing of meat products must provide sufficient evidence to demonstrate its safety and efficacy, and there is currently no universal approval. Globally, there are virtually no regulations specifically targeting ultrasound as a processing method. Its application is typically as an auxiliary technology, such as enhancing marinating or tenderizing meat. Therefore, regulation is typically carried out on a case-by-case basis, meaning companies must demonstrate that the use of ultrasound does not introduce new chemical or physical hazards, can consistently achieve the claimed technical effects, and ensures food safety.

In summary, clear legal provisions are key to the adoption of new technologies. For non-thermal technologies, future application prospects largely depend on whether the industry and academia can provide sufficient, reliable safety and efficacy data to drive regulatory bodies to establish clearer guidelines and standards.

**Table 3:** Ultrasound and UHP assisted techniques to improve meat tenderness.

References	Research Purpose	Research Methodology	Main Findings
[79]	Ultrasound + effect of sodium bicarbonate marinade on chicken breasts	WC/SC/USC Comparison	USC pickling had the highest uptake (11.1%), the lowest shear (6.99 N), significant muscle fiber breakage (MFI = 61.65), and the best overall results
[83]	Effect of ultrasound-assisted dry curing on beef protein and flavor	Beef was ultrasonicated for 90 min and then statically marinated to measure protease and free amino acids	Ultrasound accelerates protein degradation, and 90 min ultrasound + 12 h curing is equivalent to the traditional 24 h curing
[84]	Effect of ultrasound probe parameters on salt diffusion in cured meat	Different sizes of probes and distances were used to treat pork and analyze the distribution of NaCl	0.3 cm distance diffuses fastest, 0.5 cm balances efficiency and quality, and distance is the key parameter
[85]	Effect of ultrasound frequency on moisture migration and structure of pork meat	23.6–55 kHz ultrasonication of pork for analysis of moisture and muscle fiber structure	26.8 kHz sound field homogeneity, promotion of salt penetration, ultrasound disruption of muscle fiber interstitial space, and amplification test for home refrigerators
[92]	Single-frequency ultrasonic defrosting of goose meat	28/50 kHz + multi-temperature combinations	50 kHz ultrasound shortens thawing time by 57.58%, achieving hardness 173.2 N at 25 °C, protein structure stabilization, and best results
[93]	Effect of ultrasonic thawing at different frequencies on beef quality	Single/dual/triple frequency ultrasonic defrosting, determination of defrosting time, loss rate, tenderness, etc.	22 kHz single-frequency and 22/33 kHz dual-frequency defrosting with high efficiency, good tenderness, and uniform moisture distribution
[96]	Comparison of physical field thawing of livestock and poultry meat	RTT/SWT/MT/UT/IT vs	Ultrasonic thawing loss is the lowest (43% for pigs), TBARS value is reduced by 14.58% to 15.87%, and water retention and antioxidant properties are optimized
[105]	Effect of ultrasound on beef tenderness and sensory after storage	Beef was ultrasonicated at 40 kHz after storage to measure shear force and sensory characteristics	HIU reduces shear, improves tenderness, achieves better results after storage, and improved sensory odor but with slight color change
[130]	Effect of high-pressure thawing on water holding capacity and ultrastructure of pork meat	High-pressure thawing at 70–210 MPa to analyze thawing loss, protein denaturation, and electron microscopic structure	Minimal thawing losses at 140 MPa, denaturation of myosin due to high pressure, and significant contraction of muscle segments at 210 MPa
[143]	Investigating the effect and mechanism of myostatin + ultrahigh pressure to inhibit fishy odor in snakehead fish	300 MPa UHP + 25 mM carnosine treatment to analyze VOCs, TMA-N, lipid oxidation, etc.	The combination effectively inhibits the generation of fishy substances and extends the shelf life by 6 days, which is achieved through antioxidant and enzyme inactivation mechanisms
[144]	Comparison of the effects of sequential and simultaneous ultrasonic thawing on the quality of small yellow croaker (Lepomis macrocephalus)	Three-frequency sequential (TSEU)/simultaneous (TSIU) ultrasonic thawing, analysis of protein structure, texture, etc.	TSEU thawing quality is better, preserves alpha-helix structure, reduces oxidation, and is superior to TSIU and running water thawing
[145]	Ultrasound + effect of low-temperature short-term heating on protease and texture of yellow feather chicken meat	Determination of protease activity and texture by sonication at 40 kHz and heating at 55 °C	Inactivation of proteases to reduce protein degradation, improve texture, and extend shelf life
[146]	Effect of triple-frequency synchronized ultrasound on the efficiency and quality of pork curing	20 + 40 + 60 kHz ultrasound (85–150 W/L) treatment to analyze NaCl permeation, moisture, etc.	101.3 W/L sonication significantly increased NaCl content by 59.95% and improved water retention and texture
[147]	Effect of high-pressure preconditioning on the stability of pork in supercooled preservation	50–200 MPa high-pressure treatment followed by ultra-cold storage to analyze ice nucleus inhibition and protein structure	200 MPa inhibits ice nucleation, stabilizes protein structure, and prolongs freshness with ultra-cold storage
[148]	Effect of high-pressure treatment on protein and moisture in frozen storage of pork	200–400 MPa high-pressure treatment followed by freezing at for 84 days to analyze protein structure and drip loss	300–400 MPa reduces drip loss 35% and protein-water interaction dynamics affect water holding capacity
[149]	Effect of high-pressure treatment on the sodium water dynamics and structure of dry-cured hams	0.1–900 MPa treated hams analyzed by ^23^Na-NMR and TEM	600 MPa disrupts myofibrils and promotes sodium binding and release, explaining the increased saltiness of HPP hams and providing a rationale for low-sodium products
[150]	An overview of the application of HPP in salt-reduced meat products	Analyzing the effect of HPP on the functional and sensory properties and safety of salt-reduced meat	HPP improves texture and water retention and extends shelf life for salt-reduced meat products
[151]	Effect of ultrasound-assisted dry curing on the color of pork meat	Pork with different salt content was QDS dried and autoclaved to measure color parameters	High-moisture samples show increased brightness with HPP, low-moisture samples show no change, and salt substitution does not affect color
[152]	Effect of HPP and storage temperature on microbiology and oxidation of dry cured meat	600 MPa treatment of dry cured meat inoculated with *Listeria monocytogenes*, stored at 4/18 °C	600 MPa effective sterilization, storage temperature affects microbial growth, and HPP promotes oxidation with little color effect
[153]	Ultrasonic combined thawing quality of red drum fish	UT/MT/IT/UMT/UIT vs	The UMT/UIT group had intact muscle fibers and good retention of fixed water, making UMT/UIT superior to single sonication or conventional thawing

### 2.2. Intelligent Digital Integration Advancements

#### 2.2.1. IoT-Enabled Blockchain Traceability Technology

The IoT is a next-generation connectivity technology that integrates physical devices, sensors, and various objects into a unified network through internet protocols [155,156]. This system enables real-time data collection, transmission, and processing, facilitating seamless communication between objects and between humans and machines [157]. At its core, IoT relies on sensors to capture real-world status information, which is transmitted to cloud platforms or local servers for analysis and decision-making using wireless communication technologies [155,158]. The foundational components supporting IoT include sensor technology, wireless communication systems, cloud computing, and big data analytics [159].

Blockchain, by contrast, is a decentralized and distributed ledger technology that has fundamentally transformed data security and integrity protocols. Its key characteristics include immutability, transparency, and traceability [159,160]. In blockchain systems, data records or transactions are packaged into “blocks” and appended to a continuously growing digital ledger. These blocks are stored across multiple decentralized network nodes rather than on a centralized server. This architecture ensures that no single entity has unilateral control over the data, thus preserving its authenticity and preventing tampering. All network participants collaboratively validate and maintain the data integrity within the system [161].

In the meat industry, the convergence of IoT and blockchain technologies offers a robust framework for constructing efficient, transparent, and trustworthy supply chain management systems. IoT-enabled devices such as temperature sensors, humidity detectors, and GPS trackers can monitor critical environmental and product parameters across the entire supply chain—from livestock breeding and slaughtering to processing, transportation, storage, and retail—ensuring real-time quality and safety monitoring [34]. The data collected by IoT devices can be recorded on the blockchain, enabling secure and tamper-proof traceability.

Practical implementations of this integration have already emerged. For instance, in Portuguese ham production, 12 categories of data, including slaughter dates and inspection records, are uploaded to the Ethereum blockchain, allowing consumers to verify the product’s full lifecycle through hash values [162]. During chilled meat transportation, the use of IoT sensors and blockchain has reduced temperature anomaly alerts (>4 °C) by 68% [163]. Ultra-high-frequency radio-frequency identification (RFID) tags enable batch-level tracking with read rates up to 99.7%, outperforming traditional barcoding systems [164]. Furthermore, temperature data logged on the blockchain can be linked to predictive shelf-life models and used to optimize delivery routes dynamically [165]. Emerging applications also include the expansion of blockchain-integrated systems such as HFF-QCMD—originally designed for sulfonamide detection in honey—to meat products like pork, enabling full-process traceability of antibiotic usage from farm to retail [166]. Additionally, deeper integration of IoT technologies allows robotic systems in slaughterhouses to adapt cutting paths in real time with an error margin of less than 0.5 mm, thereby enhancing precision and safety [167].

Thanks to the immutable and traceable nature of blockchain, once data are uploaded, they are virtually impossible to alter, providing reliable certification of meat origin, processing conditions, and logistical history. This significantly enhances consumer trust and supports the sustainable development of the meat industry [159]. As highlighted in the work of Režek Jambrak [168], IoT has already proven its value in food processing and is expected to evolve further by integrating with emerging technologies such as non-thermal processing and 3D printing, thereby accelerating the transition of the meat industry toward a more intelligent, secure, and sustainable digital future.

Although IoT and blockchain technologies hold promise for traceability, transparency, and security in the meat industry, their large-scale adoption still faces numerous obstacles. The key technical and economic barriers are as follows. In the meat industry, the types and formats of data generated at different stages vary significantly. The absence of unified data standards can lead to information silos, hindering the effective operation of blockchain systems; IoT devices have relatively weak security and are susceptible to attacks. If data is maliciously tampered with or forged, it would directly impact the credibility of the blockchain system; additionally, the implementation of this technology requires substantial financial investment, including hardware devices (sensors, scanners, etc.), software systems (blockchain platforms, data analysis tools, etc.), and human resources (technical personnel, managers, etc.), which places significant management pressure on small and medium-sized meat enterprises. Throughout the production process, IoT and blockchain systems from different companies are involved, and if there is a lack of interoperability between them, it would affect the overall efficiency of the supply chain.

Legal and regulatory frameworks must also evolve in tandem. The application of blockchain technology involves multiple legal issues, such as data privacy and intellectual property rights. Without clear legal and regulatory frameworks, the application of blockchain technology will face uncertainty. Finally, consumer awareness and acceptance constitute the social dimension of technology adoption. Consumers’ understanding of blockchain technology directly influences their purchasing intent. If consumers lack awareness of the technology’s principles and advantages or question its security, they will be reluctant to purchase such products.

Therefore, future research and practice must not only continue to optimize the technology itself—such as establishing industry-wide data standards; specifying data collection, storage, transmission, and sharing methods; setting agreed-upon technical standards and interface specifications to ensure seamless connectivity and communication between different systems; and enhancing the security of IoT devices through identity authentication, data encryption, and access control to prevent malicious attacks—but also reduce hardware and software investments through cloud services and open-source software. The government can also provide subsidies or preferential policies to promote the adoption of this technology in businesses; the government and industry associations should strengthen the regulation of blockchain technology, establish relevant laws and regulations to clarify the rights and obligations of all parties, and enhance public awareness and trust in the technology through consumer education. Overcoming these challenges is essential to achieving the sustainable development of IoT and blockchain technology in the meat supply chain [169].

#### 2.2.2. AI-Driven Quality Prediction Technology

AI and machine learning (ML) are among the most rapidly evolving and transformative technologies in the modern technological landscape. AI refers to the discipline of simulating and extending human cognitive capabilities, with the goal of enabling machines to perform tasks traditionally requiring human intelligence—such as environmental sensing, logical reasoning, autonomous learning, planning, and predictive modeling [170,171]. AI systems are typically characterized by robust data analytics, pattern recognition, and self-learning functions, allowing them to efficiently process vast and complex datasets.

**Figure 3:**
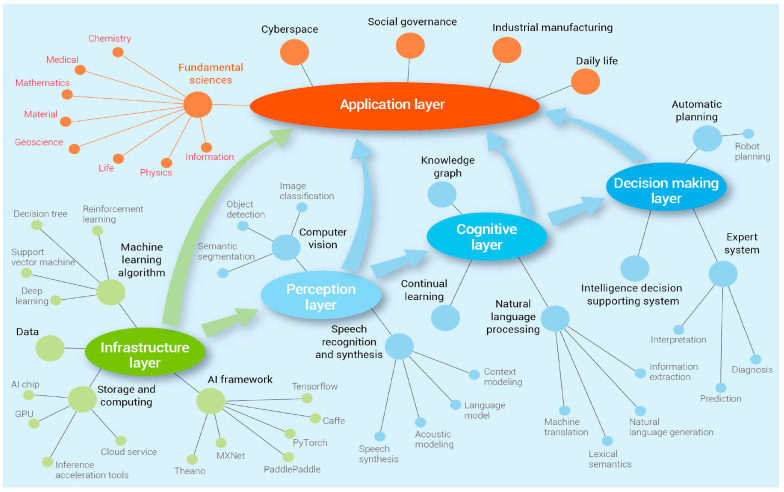
General framework of AI [171].

In the meat industry, AI has found applications across the entire supply chain—from livestock breeding and environmental management to processing, logistics, and retail [172]. As illustrated in Figure 3, an integrated AI architecture can aggregate data from farm environmental sensors, slaughtering and processing lines, cold chain logistics, and marketing channels. Through comprehensive data analysis, AI enables real-time optimization of operations. For example, AI-enabled smart farming terminals can monitor and regulate variables such as temperature, humidity, light intensity, and gas concentrations in real time, issuing alerts when critical thresholds are exceeded [173]. In processing environments, AI-powered robots and automated systems can perform repetitive, labor-intensive tasks with precision. A newly developed robotic meat factory system, for instance, achieves bone-positioned cutting path planning with ±2 mm accuracy using a modular design approach [174]. Collaborative robots adjust cutting depths using force-control algorithms to minimize bone fragmentation and preserve muscle integrity [175]. Smart knives equipped with force sensor arrays can detect resistance changes in real time, and a recurrent neural network (LSTM) is used to predict tissue lamination direction, thereby improving segmentation efficiency by 34% [21].

ML, a core subfield of AI, is a data-centric paradigm focused on the development of algorithms that enable systems to automatically learn and extract meaningful patterns from large datasets without explicit programming [176,177]. Through iterative learning, ML models continuously refine their performance and improve their capacity for accurate prediction and decision-making. ML plays a critical role in building intelligent robotic control systems, machine vision platforms, and other complex industrial applications [178,179,180,181]. However, the effectiveness of ML models heavily relies on a large amount of high-quality data for training. Insufficient or low-quality data can seriously affect the performance and generalization ability of the model, and data bias can lead to algorithm bias, resulting in poor model performance [182].

In meat processing, ML has been widely utilized for tasks such as automated defect and foreign object detection. For instance, Pushparaj combined a one-dimensional generative adversarial network (GAN) with semi-supervised learning to detect contaminants in poultry meat using a limited amount of labeled data with high accuracy [183]. Mahanti developed an impedance spectral sensing system coupled with a random forest algorithm to simultaneously monitor volatile organic compounds (VOCs) and pH levels in meat, enabling both spoilage prediction and foreign object detection [184]. Rady applied RGB imaging and ML algorithms to detect adulteration in minced meat, achieving 100% classification accuracy with a linear discriminant classifier, and an R-value of 0.98 using a regression tree model [185]. It is worth noting that when pursuing high-accuracy models, there is a risk of overfitting (which refers to the phenomenon where a model performs well on training data but poorly on new data it has not seen before). To avoid this situation, strict validation strategies such as cross-validation, data augmentation, and regularization can be adopted to improve the model’s generalization ability [186].

Deep learning (DL), a specialized subset of ML, has emerged as one of the most active research areas in AI [171]. It involves constructing multi-layered neural network models capable of hierarchical feature extraction and representation learning, enabling the resolution of complex nonlinear problems [187]. DL has achieved notable success in fields such as image recognition, speech processing, and natural language understanding, and it is now being applied to specific meat industry challenges [188,189,190]. For example, a globally calibrated DL model based on eight datasets achieved cross-species prediction of intramuscular fat (IMF) content and pH in beef, lamb, and venison, outperforming traditional models by 12–15% in accuracy [191]. Bai et al. developed a dual-modal X-ray–visible imaging system with a 90% detection rate for bone fragments embedded in meat products [27]. A fusion system combining hyperspectral imaging (HSI) with convolutional neural networks (CNNs) achieved 94.3% accuracy in meat freshness detection [192]. Ahmed’s team established a PLS-DA model based on hyperspectral data (400–1000 nm range), which improved beef grading accuracy to 92.8%. Compared to manual grading, the system increased processing speed by 15-fold and improved grading consistency by 40% [193]. A vision-guided robot based on the ResNet-50 architecture achieved a cutting accuracy of ±0.3 mm for pork segmentation and dynamically adjusted parameters through real-time feedback, increasing raw material utilization by 12% [194].

DL models are particularly complex, and their decision-making processes are often difficult to understand and accept. To improve transparency, explainable AI (XAI) technologies have been developed to make the decision-making processes of models more transparent [195]. Additionally, if the training data fail to adequately represent the diversity of meat types or specific processing conditions, DL models may also exhibit bias, leading to unfair or erroneous decisions [196]. This also involves considerations across legal, ethical, and social dimensions.

Training and deploying AI models present significant challenges. It requires robust computational infrastructure support [197], including high-performance computing clusters, GPU servers, big data storage systems, stable and reliable network connections, efficient data transmission channels, and ongoing maintenance and upgrades. For institutions or individuals with limited resources, these infrastructure requirements entail significant cost investments and technical barriers.

The widespread application of AI has also had a profound impact on the labor market [198]. On one hand, AI has automated many repetitive, tedious, or dangerous tasks, leading to job losses for some workers; on the other hand, AI has also created new employment opportunities, such as AI engineers and data scientists. Therefore, it is essential to strengthen skill training for workers to enhance their ability to adapt to new technologies [199]. Additionally, governments and society should provide necessary social security measures to assist unemployed individuals in smoothly transitioning to new job roles.

In summary, AI-driven integrated technologies are accelerating the transformation of the meat industry from labor-intensive to data-intensive and intelligence-driven paradigms. Through real-time data acquisition, intelligent analytics, decision-making, and automated execution, these technologies significantly enhance production efficiency, product quality, and food safety. Furthermore, they optimize resource utilization, minimize environmental impact, and increase transparency and traceability across the entire value chain, aligning with consumer demands for high-quality, safe, and sustainable meat products. This transformation process must be strictly managed, requiring a thorough understanding and resolution of challenges such as data limitations, model overfitting and validation, high equipment costs, algorithm transparency, potential algorithmic bias risks, and the social impact of changes in the labor market structure. Only through rigorous technical development, strict ethical considerations, and feasible labor planning can AI be fully utilized in the meat industry to truly serve humanity and promote the industry’s healthy development.

### 2.3. Emerging Resource Utilization Breakthroughs

#### 2.3.1. Cell-Based Cultured Meat Production Technology

Cell-based cultured meat, also referred to as lab-grown or in vitro-cultured meat, represents a disruptive innovation that is fundamentally reshaping conventional meat production systems [200]. The core of this technology lies in the application of cell and tissue engineering principles to culture animal-derived cells—primarily muscle and fat cells—in a controlled in vitro environment, resulting in a product that closely mimics the structural and nutritional characteristics of conventional meat [201]. The final product is an organized assembly of muscle cells and their biochemical derivatives, designed to serve as a sustainable and potentially healthier alternative to animal-derived meat [202]. Compared to conventional animal agriculture, cell-cultured meat offers significant environmental advantages. It has the potential to drastically reduce greenhouse gas emissions, land usage, and water consumption associated with intensive livestock farming, while also mitigating pollution and offering a reliable and safe protein source [203].

The production of cultured meat typically involves three major steps: the isolation and expansion of target cells (e.g., stem cells or satellite cells), large-scale bioreactor cultivation to induce differentiation into muscle fibers and adipocytes, and structural assembly into a three-dimensional meat-like product [204]. For example, studies have demonstrated that porcine muscle stem cells can be expanded and differentiated in vitro to form functional muscle fibers with characteristics similar to those of natural meat tissue [205]. According to experimental data, approximately 5.3 × 10^4^ muscle satellite cells (MuSCs) can be isolated from one gram of neonatal pig muscle tissue but must undergo a 10^6^–10^7^-fold expansion to yield 100 g to 1 kg of cultured meat [205]. However, scaling up laboratory-scale cell culture efficiently and economically to industrial production scale is a key challenge currently facing the industry [206].

To improve cell expansion efficiency, several biochemical strategies have been explored. Valproic acid (VPA), for instance, has been shown to enhance porcine MuSC proliferation by approximately 34% while preserving myogenic differentiation potential [207]. Flavonoids such as 3,2′-dihydroxyflavone (10 μM) have also been found to activate signaling pathways that upregulate PAX7 expression by 60%, resulting in a 34% increase in total cell number [208]. Additionally, the IMEM medium developed by Fiona outperformed standard DMEM by achieving a cell proliferation rate of up to 2.583 doublings per day (R^2^ = 0.975) [209]. Nevertheless, the high cost of culture media remains a major bottleneck for industrialization.

The commercial viability of cultured meat hinges on several key technological advancements: the optimization of culture media to reduce costs, the enhancement of growth efficiency, the control of metabolic by-products, and the achievement of high-density cell expansion [210]. Although substantial progress has been made in large-scale myoblast expansion and directed differentiation, however, the industrialization process still faces numerous obstacles: There are high production costs, particularly the cost of cell culture media (containing animal serum, growth factors, etc.), which are extremely expensive and constitute the primary constraint on product pricing [211]; large-scale bioreactors require continuous and substantial energy inputs, with environmental impacts and operational costs that cannot be ignored; scaling up laboratory-scale cultures to industrial-scale production that meets market demand (e.g., cell uniformity, nutrient delivery, and waste removal) presents significant challenges [206]; and public awareness, attitudes, and purchasing intentions toward new technologies are also important influencing factors [212]. Additionally, a unified and clear legal regulatory framework for approving such products has yet to be established [213].

Future research will likely prioritize improving cell proliferation rates, developing economically viable and chemically defined media, and designing scalable bioreactors capable of supporting high-density cultures. Moreover, enhancing the sensory characteristics of cultured meat—such as texture, juiciness, and flavor—will be essential for consumer acceptance. Public engagement and transparent communication will also play a critical role in fostering societal acceptance and encouraging the responsible development and dissemination of this emerging technology [214].

#### 2.3.2. Three-Dimensional Bioprinted Tissue Fabrication Technology

Three-dimensional (3D) printing, a subset of additive manufacturing (AM) technology, is based on the principle of constructing complex geometries by depositing materials layer by layer according to digital 3D model data [215]. In recent years, this technology has demonstrated substantial potential in food science and engineering, particularly within the meat industry, where it enables revolutionary innovations in product customization, structural optimization, and the development of novel meat analogs such as cultured meat.

A major application of 3D printing in the meat sector lies in the creation of personalized food products. By precisely adjusting printing parameters and material formulations—often referred to as “food inks” (e.g., plant-based proteins, microalgae derivatives, or other functional compounds)—it is possible to fabricate meat products with customized shapes, textures, nutrient profiles, and visual characteristics [216,217]. This high degree of control supports flexible product design and targeted nutritional functionality. For instance, researchers have successfully printed cultured meat with a steak-like structure by precisely modulating the ratio of muscle to fat, thereby enhancing taste, texture, and thermal stability during cooking [218]. Additionally, edible porous microcarriers have enabled the bioprinting of marbled, cell-cultured fish meat by facilitating the large-scale production of integrated muscle–fat microtissues [219]. Similarly, a composite system based on pea protein and κ-carrageenan formed a double-network gel at pH 5.0 with an elastic modulus (G’) of 1.2 × 10^4^ Pa, closely resembling the textural properties of real beef [220].

However, the production volume and formulation of ideal personalized products are constrained by various factors. The materials used in 3D printing must possess specific rheological properties, such as shear thinning, appropriate viscosity, and yield stress, to ensure printability and structural integrity [221]. This means that printing materials are typically viscous, uniform pastes, limiting the types of ingredients and formulation options available. Additionally, to ensure the precision and stability of the printed structure, the formulation must be finely adjusted, further limiting its flexibility. Meanwhile, how to use 3D printing technology to achieve textures, flavors, aromas, and colors similar to or better than those of traditional foods, while ensuring the stability and bioavailability of nutrient-active components (such as vitamins and probiotics) during processing, remains a key issue to be addressed [222].

Beyond personalization, 3D printing plays a vital role in improving the structural stability of both traditional and alternative meat products. A recurring challenge in the 3D printing of food materials is insufficient structural support, which can lead to product collapse during fabrication or post-processing. Several strategies have been developed to overcome this issue. For example, localized laser-assisted heating has been employed to enhance the gelation and mechanical integrity of printed minced fish, significantly improving shape fidelity and gel strength [223]. Another effective approach involves optimizing formulation parameters using statistical experimental design techniques, such as the Box–Behnken design. In the case of 3D-printed minced shrimp, such optimization significantly improved both print accuracy and structural robustness [224].

It is worth noting that 3D printing technology is not limited to innovative products; it also holds great potential for transforming existing traditional meat products. For example, it can allow for personalized customization by tailoring the shape, size, and fat-to-lean ratio of meat products according to consumer preferences. For instance, it can produce easy-to-chew minced meat products for elderly individuals with dental issues, or turn high-protein, low-fat meat products [225] into more value-added meat products, reducing resource waste [103]. It can also combine different types of meat, vegetables, and spices to print meat products with unique flavors and nutritional value.

Looking ahead, the large-scale application and commercial viability of 3D printing technology in the meat industry still face significant challenges. Key bottlenecks include currently slow printing speeds, limiting the efficiency of large-scale production (production capacity constraints); strict requirements for rheological properties, limiting the range of raw material options and increasing the complexity of formulation development; and the challenge of replicating the sensory experience of traditional meat (texture, taste, etc.) while ensuring the nutritional value of the final product.

Despite this, 3D printing is catalyzing a transformative shift in meat production through its capabilities in flexible material utilization, structural engineering, and process control. It offers substantial potential for meeting individualized nutritional requirements, facilitating the production of sustainable cultured meat, and enhancing the structural and functional properties of meat and meat alternatives. As such, this technology contributes not only to product innovation and diversification but also to the long-term efficiency and sustainability of the meat industry.

## 3. Industrial Application Pathways

### 3.1. Application in Intelligent Centralized Food Processing System Development

Central kitchens, also referred to as catering distribution centers, represent an emerging organizational model in the food industry, originally introduced from developed countries such as the United States and Japan. These facilities operate within dedicated premises equipped with specialized machinery and are primarily responsible for the centralized pre-processing and preparation of fresh food materials. The processed products—either finished or semi-finished—are subsequently distributed via cold or hot chain logistics to end-users including restaurant chains, hotels, industrial canteens, and large-scale supermarkets [226,227].

As a critical component of the modern food service infrastructure, the core objective of the central kitchen model is to enhance operational efficiency, reduce production costs, and ensure food quality and safety through standardized, large-scale, and centralized production methods [228]. In the context of the meat industry, the effectiveness and consistency of products produced within central kitchens are closely linked to the processing technologies employed, given the complexity of meat preparation, which involves multiple stages such as raw material handling, precision cutting, thermal processing, and preservation.

To maximize the functionality and competitiveness of central kitchens, the integration of advanced and emerging technologies—such as intelligent cutting systems, ultrasound-assisted processing, and HPP—has become increasingly important. These technologies not only improve processing throughput and product consistency but also reduce operational costs, minimize material loss, and enhance food safety. Moreover, the synergistic application of multiple smart technologies can further amplify efficiency and drive the transformation of the meat industry toward a more intelligent, automated, and sustainable production paradigm.

#### 3.1.1. Industrial Deployment of Intelligent Cutting Systems

Meat cutting is an inherently complex operation due to the softness of biological tissues and the heterogeneous composition and morphology of meat. During manual processing, operators typically rely on the integration of multiple sensory modalities—such as vision, touch, and even olfaction—to skillfully perform accurate and efficient cutting tasks [229]. In robotic meat processing, multimodal sensing systems are employed to replicate this multisensory perception and enable intelligent, adaptive operation [29]. However, differences between individual carcasses and between batches (such as fat distribution, muscle texture, size, and shape) pose a challenge to the generalization capabilities of sensing systems and algorithms, requiring robots to be capable of adapting to different cutting tasks [230].

Specifically, visual sensors simulate human vision to acquire spatial and structural information from meat samples and, when coupled with advanced image processing algorithms, facilitate precise control and dynamic target tracking [231]. Simultaneously, force sensors detect variations in cutting resistance and tissue elasticity, providing real-time feedback that allows robotic arms to adjust cutting trajectories dynamically [232]. However, the visual system is susceptible to changes in lighting, surface moisture, bloodstains, and carcass variability, necessitating the implementation of strict online calibration procedures [233]. Force sensors are susceptible to contamination and drift in the humid and greasy environment of meat processing, necessitating frequent calibration and maintenance to maintain accuracy. Force measurements at the cutting contact points are also interfered with by the sharpness of the tools and the viscoelasticity of soft tissues.

**Figure 4:**
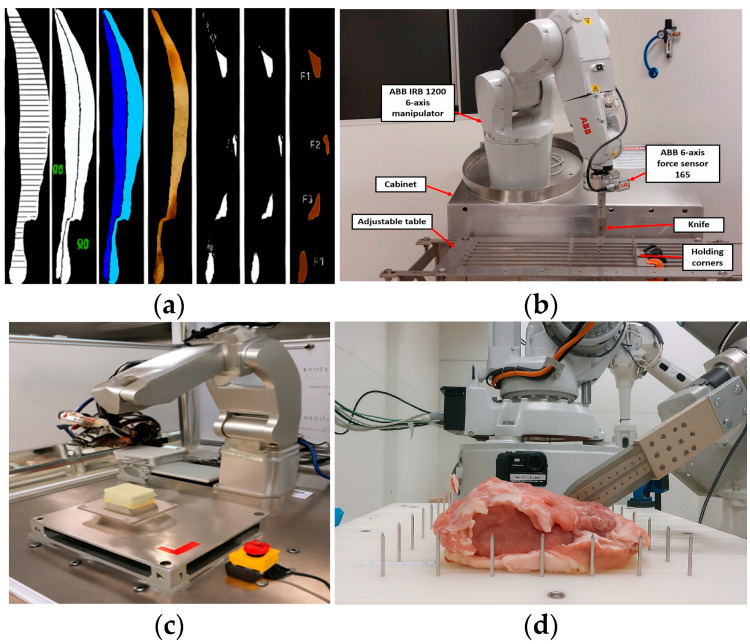
Visualization of the whole process of fin segmentation (**a**) [36]; hardware setup to verify the feasibility of haptic sensing in robotic red meat cutting (**b**) [37]; experimental hardware configuration (**c**) [20]; demonstration of a real-world scenario of meat cutting by a smart knife in collaboration with a robot (**d**) [21].

Numerous studies have validated the effectiveness of vision-based systems in meat segmentation and localization. For example, as shown in Figure 4a, Azarmdela, Mohtasebi et al. [36] utilized machine vision to segment salmon fins and extract cutting points across varying fish sizes, significantly improving processing accuracy. Zhao, Hao, and Syahputra et al. [19] developed an ICNet that achieved 97.68% segmentation accuracy and a mean intersection-over-union (MIoU) of 88.47% for real-time sheep carcass analysis. Although ICNet demonstrated strong performance, its accuracy declined slightly in structurally complex regions such as the neck and rib area; lightweight models may be adopted subsequently to balance computational efficiency and model precision. Similarly, Huang [38] applied an improved ResNet-50 model to recognize different anatomical regions in pork, achieving 94.47% accuracy. This approach enables robotic systems to execute cuts at predefined positions and angles, reducing manual intervention and enhancing yield. Esper and Romanov et al. [18] designed a multi-view data acquisition platform using six Intel RealSense D415 depth cameras (Intel Corporation, Santa Clara, CA, USA) to collect RGB texture and 3D depth data of meat surfaces. By integrating multimodal data through a trained model, they achieved accurate identification of pork epidermis, muscle fibers, and adipose tissues, significantly advancing automation in meat cutting lines.

Force sensing technologies also play a critical role in robotic cutting. Aly et al. [37] attached force sensors to a six-axis robotic arm to perform 20 mm-deep steak cuts (Figure 4b), revealing that cross-correlation coefficients between force signatures of muscle and fat tissues ranged from 80% to 97%, providing a theoretical basis for dynamic trajectory optimization. Maithani et al. [39] proposed “Exoscarne”, a human–robot collaborative cutting system, which integrates force sensors via impedance control into a KUKA LWR robot. The system reduced operator wrist strain by approximately 30%, demonstrating practical ergonomic benefits. Takács et al. [234] introduced a novel force control method using strain gauges and magnetic encoders to enable a smart gripper to perform precise cuts. This gripper can handle both large, heavy items (e.g., pork legs, organs) and delicate, soft structures (e.g., tracheas) with high accuracy.

During meat cutting, soft tissues undergo a combination of elastic deformation, plastic deformation, and eventual fracture [235]. The timing of incision critically affects the quality of the cut surface, with premature or delayed cuts potentially leading to rough edges or uneven cross-sections [29]. Real-time monitoring of cutting force dynamics and synchronized adjustment of tool parameters can significantly enhance cutting quality and efficiency. For instance, as shown in Figure 4c, the Kato team [20] implemented a time-delay neural network (TDNN) to classify the deformation behavior (elastic or plastic) of flexible meat tissues. Based on this classification, a multi-degree-of-freedom robotic arm performed cutting actions while dynamically optimizing its trajectory. The experimental results demonstrated consistently high cutting success rates across varying speeds.

Beyond vision and force sensors, electromagnetic wave sensors have also been integrated into robotic meat cutting. Mason et al. embedded smart blades equipped with electromagnetic sensors into a robotic arm, enabling real-time feedback on the tool–meat interaction [236]. In pork cutting experiments (Figure 4d), this system exhibited a maximum error of 1.78% and an average depth error of 7.66 mm (±1.45 mm), reflecting strong overall performance [21]. Roer [237] further evaluated the smart knife in comparison with three other robotic cutting tools. Although it did not achieve the highest composite score, the knife’s built-in sensors allowed for real-time monitoring of contact force and cutting depth, reducing detection error to 2.92% and depth error to 7.22 mm, thereby enhancing both accuracy and operational safety. For integrated sensor systems, strict food hygiene standards must be followed. Food-grade sensor housing materials that are easy to clean and disinfect should be used to prevent contamination and meet regulatory requirements [21,238].

While robotic cutting technology demonstrates advantages in terms of precision, reducing manual labor, and performing specific tasks, comprehensive efficiency (including total processing time, output rate, etc.) still requires careful evaluation compared to experienced operators. This evaluation should consider a comprehensive assessment of indicators such as physical ergonomics, cognitive ergonomics, health and safety, and operational efficiency. In the future, by collecting data from actual workstations, the potential of robotic systems can be systematically assessed to ultimately improve production efficiency and enhance the work environment [239].

#### 3.1.2. PEF Integration in Commercial Meat Processing Lines

PEF technology has demonstrated significant potential in improving meat tenderness and accelerating the curing process in industrial settings [240]. Experimental studies have revealed that PEF treatment induces considerable changes in meat microstructure, with notable enhancements in tenderness, particularly when used in conjunction with freeze–thaw processing. For instance, application of PEF to both fresh and freeze–thawed beef muscle significantly improved tenderness, with scores increasing from 24.73 to 42.01, especially for freeze–thawed samples [63,237]. In the context of venison processing, both low-intensity (LPEF, 2.5 kV) and high-intensity (HPEF, 10 kV) PEF treatments enhanced the quality of wet- and dry-cured products. High-intensity PEF treatment improved tenderness by 9% and accelerated the drying rate by 6% [62]. Additionally, PEF at moderate conductivity levels (6–9 mS/cm) significantly reduced the firmness of beef ribs, whereas lower or higher conductivities required extended cooking times to achieve similar effects [57]. PEF also facilitates faster and more efficient marination. One study demonstrated that PEF pretreatment increased NaCl uptake and diffusion coefficients by approximately 58.5%, enhancing the water-holding capacity, color, and tenderness of the meat [55]. Collectively, these findings underscore the potential of PEF as a scalable, non-thermal technique for improving processing efficiency and meat quality in commercial meat production lines.

#### 3.1.3. Ultrasonic Processing Solutions for Industrial-Scale Production

Ultrasound-assisted processing has emerged as an efficient, non-thermal technology capable of improving multiple aspects of meat production at an industrial scale. This technique not only accelerates thawing and reduces the impact of ice crystal formation but also enhances marination efficiency and improves meat tenderness [144,241].

For instance, Guo, Yang, and Ma et al. [242] demonstrated that a 400 W ultrasonic treatment significantly reduced thawing and cooking losses while preserving muscle fiber integrity, thereby improving both thawing efficiency and the quality of frozen yak meat. A comparative study of five thawing methods [153] showed that ultrasound combined with microwave thawing (UMT) or far-infrared thawing (UIT) stabilized the secondary and tertiary protein structures, maintained muscle fiber architecture, and minimized protein denaturation and water migration.

Furthermore, a study comparing conventional thawing techniques (e.g., room temperature, static water) with physical field-assisted thawing methods (e.g., microwave, ultrasound, infrared) confirmed that ultrasound-assisted thawing better preserved meat color and texture while effectively inhibiting oxidative damage to proteins and lipids, ultimately yielding the best overall thawing results [96]. A combined ultrasound and mild heat pretreatment at 40 kHz, 0.2 W/cm^2^, and 55 °C for 15 min improved protease inactivation and extended shelf life in chicken meat through synergistic effects [145].

In aquatic species, three-frequency sequential ultrasonic defrosting (TSEU) proved most effective for small yellow croaker, offering the lowest thawing loss and the highest similarity to fresh samples [144]. For goose meat, single-frequency ultrasound-assisted thawing at 50 kHz and 25 °C significantly reduced thawing time and loss, while also altering the protein structure to produce more tightly packed and irregular myogenic fibers [92]. Wu, Qiu, and Guo et al. [93] evaluated single-, dual-, and triple-frequency ultrasound modes and found that 22 kHz single-frequency and 22/33 kHz dual-frequency configurations delivered the best results in terms of defrosting efficiency, water-holding capacity, and beef tenderness. Additionally, Chen, Sun, and Xuan et al. [243] developed a machine learning-based ultrasonic monitoring model for beef thawing, enabling non-destructive quantitative analysis with significantly higher prediction accuracy than traditional methods, thereby improving food safety and quality control in industrial processing environments.

Ultrasound-assisted marination has also demonstrated remarkable potential for improving processing speed and flavor enhancement. Studies have shown that ultrasound accelerates salt diffusion and reduces marination time by up to fourfold compared to conventional methods [84]. High-intensity ultrasound (HIU) at 40 kHz and 11 W/cm^2^ for 60 min improved the tenderness of marinated beef [105], while triple-frequency synchronized ultrasound (20 + 40 + 60 kHz) at 101.3 W/L significantly promoted NaCl penetration, enhancing the overall curing quality of pork [146].

In poultry applications, synergistic curing using ultrasound and sodium bicarbonate (USC) significantly improved marination speed, water retention, and tenderness in chicken breast meat, making it an effective strategy for industrial curing [79]. Combined ultrasound (40 kHz, 140 W) and pressure (UPV) treatment further enhanced curing performance by disrupting muscle fiber structures through cavitation, increasing water-binding capacity, and promoting brine diffusion through dynamic osmotic pressure modulation. These changes ultimately reduced water loss and improved tenderness [244].

Moreover, ultrasonic treatment at 26.8 kHz enhanced the water-holding capacity of pork by accelerating NaCl diffusion, converting free water to bound water, and improving tenderness via disruption of the fiber matrix [85]. In dry-cured beef processing, ultrasound at 400 W and 25 kHz for 90 min effectively loosened protein structures, increased free amino acid content, and significantly enhanced flavor development [83].

Collectively, these findings highlight the versatility and scalability of ultrasound-assisted processing as a promising approach for improving the efficiency, sensory quality, and safety of meat products in industrial production environments.

#### 3.1.4. HPP Implementation in Modern Meat Facilities

HPP has demonstrated outstanding performance in meat marination, microbial inactivation, and shelf-life extension. As a non-thermal sterilization technology, HPP not only enhances food safety but also maintains the nutritional and sensory qualities of meat products. For example, Cava [152] reported that treatment at 600 MPa for 8 min effectively reduced *Listeria monocytogenes* to within regulatory limits, extending product shelf life to a minimum of 30 days. Similarly, a combined treatment of ultra-high-pressure (300 MPa for 900 s) and sarcosine in fish fillets significantly inhibited the formation of off-odor compounds such as trimethylamine, effectively prolonging shelf life [143].

Ferrini et al. [151] integrated the quick dry slicing (QDS) process with HPP (500–600 MPa for 7 min) and successfully produced low-sodium, safe, and color-stable dry-cured meat products. Li et al. [245] found that pulsed-pressure curing (PPC) significantly enhanced curing efficiency, improved beef tenderness, and elevated sensory attributes. Furthermore, HPP has been shown to influence the sodium and water distribution in dry-cured hams. Treatment at 600 MPa resulted in tighter muscle fiber alignment and increased sodium content, whereas 900 MPa reduced water-holding capacity—together intensifying the perception of saltiness without raising actual sodium levels [149].

In sausages, HPP treatment at 100–200 MPa reduced the number of viable microorganisms, decreased cooking losses, and improved both texture and flavor. These effects were particularly beneficial in compensating for sensory deficiencies in low-sodium formulations [246]. Nuygen et al. [150] further demonstrated that combining HPP with functional ingredients (e.g., starch, κ-carrageenan) or alternative salt sources (e.g., potassium chloride, KCl) in sausage production successfully reduced sodium chloride levels, offering a promising strategy for developing low-sodium, heart-healthy ready-to-eat meat products.

HPP is also gaining traction in freezing and thawing applications due to its positive effects on protein structure, water retention, and microbial stability. For instance, beef treated at −35 °C and 650 MPa for 10 min showed post-thaw color closely resembling that of fresh meat and significantly reduced microbial loads, highlighting the feasibility of high-pressure cryopreservation for long-term storage [247]. Another study exposed pork to 200–400 MPa at 5 °C for 4 min, followed by freezing at −20 °C for 84 days. The results showed that high pressure induced protein structural changes and improved water retention without significantly altering the overall water-holding capacity [148].

Moreover, thawing pork at 140 MPa for 29 min resulted in minimal thawing loss, moderate ice crystal melting, and the least structural disruption, representing optimal thawing conditions [130]. At 200 MPa, high-pressure treatment further promoted inter-fiber moisture distribution, inhibited ice crystal formation, and enhanced thawed pork quality under low-temperature conditions. However, it is necessary to further optimize pressure parameters to maximize quality retention and energy efficiency [147].

With the continuous deployment of cutting-edge technologies, the meat processing industry—especially centralized kitchen systems—is moving toward enhanced automation, intelligence, and sustainability. The integration of advanced methods such as intelligent cutting, ultrasonic assistance, PEF, and HPP is expected to significantly improve processing efficiency, ensure product safety, and enhance overall meat quality. These synergistic innovations present broad prospects for the future development of the meat industry, which is poised to meet emerging demands while embracing opportunities for high-tech transformation.

#### 3.1.5. Sustainability Challenges for Central Kitchens Enabled by Technology

The central kitchen model, leveraging automation and advanced technology, is redefining efficiency and safety standards in the food supply chain. However, while this centralized, large-scale model delivers benefits, it also faces inherent challenges. Energy consumption is a critical factor, as large-scale centralized production inevitably increases energy demand, and actual electricity consumption during production may exceed estimates in the literature. Therefore, it is essential to strictly control temperature and energy consumption during the operation of large-scale equipment, enhance the energy efficiency of electrical appliances, reduce operational costs, and minimize environmental impact. Advanced equipment such as intelligent cutting systems, ultrasonic devices, and HPP systems feature complex structures with stringent maintenance requirements and specialized expertise. Any malfunction can result in high repair costs and disrupt overall efficiency. For equipment-intensive systems, effective maintenance strategies must be designed and professional maintenance teams established to extend equipment lifespan and reduce the risk of unexpected downtime [248].

The initial investment in a central kitchen is high, involving significant expenditures on advanced specialized equipment, strict environmental control systems, facilities, processing lines, and cold chain logistics fleets. Initial investments can be made in collaboration with suppliers through equipment leasing or modular solutions, with phased expansion of scale. Application in small-scale or decentralized structures is limited, such as in remote areas where small businesses face high distribution costs (logistics, energy consumption, and time), which offset the advantages of centralized production and make it difficult to meet highly personalized, small-batch, and on-demand requirements. Mobile kitchens or similar solutions can be adopted to adapt to the unique needs of different regions.

In future development, it is essential to continuously integrate smart technologies to optimize core processes. Additionally, innovative approaches such as modular design and distributed small-scale centers should be explored to balance economies of scale with sustainability, thereby maximizing the potential of the central kitchen model.

### 3.2. Application in Intelligent Cold Chain Logistics System Optimization

Cold chain logistics for meat refers to a comprehensive technological system that ensures the quality and safety of meat products during storage and transportation by maintaining a controlled low-temperature environment. Although temperature regulations for cold storage exist in many regions, non-compliance during transportation and retail stages—including temperature fluctuations and improper storage—continues to compromise meat quality and shorten shelf life. According to the latest report from the World Health Organization in 2024, 600 million people worldwide (almost 1 in 10) fall ill each year due to contaminated food, with 420,000 deaths. Children under the age of five bear 40% of the burden of foodborne diseases, posing a serious threat to public health, economic stability, and environmental sustainability [249].

Given the interconnected nature of cold chain logistics, each node—from slaughter to consumer—must be systematically managed and technologically optimized to maintain freshness and minimize spoilage [250]. Among key factors, temperature control remains critical. In low-temperature environments, the application of high-voltage electrostatic field (HVEF) technology has proven effective in inhibiting microbial growth, delaying spoilage, and enhancing meat quality. For instance, HVEF treatment significantly increased the moisture content of pork and improved the water-holding capacity and tenderness of muscle proteins during cryogenic storage [251]. Another study demonstrated that freshly slaughtered pork treated at 12.0 kV and stored at −1.0 ± 0.5 °C exhibited enhanced structural integrity of muscle fibers and greater water retention, highlighting its suitability for cold chain logistics [252].

Furthermore, the synergistic combination of electrostatic fields (EF) with supercooling (SC) technologies has shown promise in extending shelf life by inhibiting enzymatic and microbial activity while regulating ice crystal formation [253]. An advanced integrated approach combining HVEF pretreatment, cinnamaldehyde nanoemulsion (CNE) release, and oxygen-enriched modified atmosphere packaging (MAP) was shown to effectively suppress microbial proliferation and oxidative degradation in pork fat, while preserving meat color and texture—making it a viable solution for refrigerated pork storage [254].

In parallel, blockchain technology is revolutionizing cold chain transparency and reliability. When integrated with the IoT, blockchain enables immutable data tracking throughout the entire meat supply chain—from farm to fork. Arvana proposed a multilayered blockchain-based traceability system covering all stages from breeding to marination, allowing users to access detailed production and logistics data by scanning a product’s QR code [162]. Similarly, Kour utilized IoT sensors to collect real-time data on temperature, humidity, and pathogens across processing and transport phases, which are uploaded to blockchain via smart contracts, enabling seamless traceability across farming, processing, logistics, and retail nodes [255].

In the future, intelligent meat logistics systems will incorporate automated thresholds at each supply chain stage—for instance, rejecting batches that exceed microbial limits at the processing stage or halting transport if temperature or humidity deviates beyond tolerance. AI and big data analytics can be employed to dynamically optimize transport routes, reduce fuel consumption, lower logistics costs, and minimize carbon emissions, thereby contributing to a more sustainable cold chain network [256].

Advanced machine vision technologies are increasingly utilized in real-time inspection and monitoring of meat products. These systems can extract surface texture patterns, assess fat distribution, and determine freshness grades to identify physical damage or microbial spoilage during transport [257]. For example, X-ray computed tomography (CT) has been applied to detect fish bones with classification accuracies of 100%, 98.5%, and 93.5% for large, medium, and small bones, respectively [258]. This imaging principle based on density differences can be generalized to the non-destructive detection of foreign materials in various meat types, including bone fragments and splinters.

Additionally, hyperspectral imaging combined with deep learning has enabled the development of semi-supervised hybrid models for detecting contaminants on raw chicken surfaces, achieving 96.5% accuracy without requiring extensive data annotation [259]. Another study introduced a hybrid spoilage monitoring system that integrates an electronic nose (e-nose) with computer vision. This system provides real-time detection of spoilage-inducing microorganisms and classifies meat freshness with an accuracy of 0.85 [260]. Meanwhile, infrared spectroscopy, based on molecular vibration analysis, has emerged as a reliable, non-destructive tool for assessing biochemical changes associated with meat spoilage [261].

By integrating sensor networks, IoT, and wireless communication technologies, real-time monitoring of temperature, humidity, and freshness is achievable throughout the logistics process. These systems also allow predictive modeling of shelf life via continuous data analysis, reducing dependence on manual inspections [262]. For example, the Multi-dimensional Information Sensing and Monitoring System (MISS) [263] can detect abnormalities such as temperature fluctuations, cargo displacement, or packaging breaches during transit. By combining visual and sensor-based inputs, it issues real-time alerts to ensure cargo safety and maintain product integrity throughout the cold chain.

However, the widespread application of these advanced technologies also faces practical challenges. The initial investment and maintenance costs of systems such as blockchain, AI, and ML are relatively high. Establishing a robust cold chain logistics system can provide companies with references through analysis, identifying investment hotspots, and potential risks, thereby enhancing corporate profits. Traditional cold chain logistics systems face numerous challenges in terms of integration. Traditional systems often struggle with traceability issues and unclear accountability, failing to adequately protect consumer rights; they may also use different data standards and formats, with each operating independently and lacking effective interoperability, leading to information silos; and they have limitations when handling large volumes of real-time data and intelligent monitoring [264].

For small-batch meat products, logistics costs are relatively high, necessitating optimized transportation and storage solutions to reduce costs; for large-scale meat products, efficient warehousing, transportation, and distribution capabilities are required. Insufficient infrastructure or poor management can lead to food waste and safety issues [265]. Different types of meat products have varying requirements for environmental conditions such as temperature and humidity, necessitating the selection of appropriate cold chain solutions based on product characteristics. Overcoming these challenges is crucial for the widespread commercial application of innovative technologies and the realization of a truly comprehensive meat cold chain.

### 3.3. Application in Multi-Source Data Fusion Based Freshness Monitoring

Freshness evaluation represents a critical focus within the domain of food safety, particularly for quality control of highly perishable products such as meat. Conventional assessment methods—such as sensory analysis, microbiological testing, and physicochemical measurements—are often time-consuming, destructive, and exhibit limited sensitivity. With the advancement of analytical technologies, emerging approaches including fluorescence sensing, colorimetric sensor arrays, intelligent packaging, and ML have garnered significant attention. These innovations offer enhanced accuracy, rapid response, and non-destructive capabilities, thereby addressing the limitations of traditional techniques and becoming increasingly prominent in the field of freshness monitoring.

#### 3.3.1. Smart Tag-Based Freshness Monitoring System Deployment

Smart tags, with core functionalities including real-time monitoring, information digitization, and supply chain optimization, are revolutionizing quality control and traceability in the food industry, serving as intelligent interfaces between producers and consumers [266,267]. Smart packaging technologies enable real-time freshness monitoring through the integration of sensors and visual indicators. For example, Lin developed a pathogen-responsive smart packaging system in which cinnamaldehyde-loaded nanofibers release antimicrobial agents upon detecting *E. coli* O157:H7 enzymes, thereby enhancing beef safety without compromising sensory attributes and addressing the volatility of cinnamaldehyde [268]. Wang designed an acid-compatible intelligent packaging system for the preservation of acidic foods, demonstrating significant efficacy in prolonging the shelf life of poultry products through pH-responsive material engineering [269]. Zhang developed a pH-indicator film based on dragon fruit peel pectin, cassava starch, and natural dyes (cyanidin and alizarin) for monitoring pork freshness [270]. Additionally, a bilayer film with integrated antibacterial properties and freshness indication capabilities was fabricated using electrospinning, enabling simultaneous preservation and real-time monitoring of meat products [271].

#### 3.3.2. IoT-Integrated Sensor Network for Real-Time Freshness Assessment

Advanced sensing technologies—such as electronic tongue (ET), electronic nose (E-nose), near-infrared spectroscopy (NIRS), HSI, low-field nuclear magnetic resonance (LF-NMR), and ultrasonic detection systems—have been widely adopted for food quality monitoring applications [257,272,273]. These approaches have demonstrated significant potential for real-time freshness evaluation and spoilage detection in meat products [274,275,276].

Colorimetric sensor arrays detect volatile organic compounds (VOCs) produced during spoilage by monitoring visible color changes, thereby enabling rapid freshness identification. The E-nose simulates the human olfactory system to recognize spoilage-related volatiles, while HSI captures spectral signatures of meat and utilizes ML algorithms to assess freshness levels. Mid-infrared (mid-IR) spectroscopy, when combined with predictive modeling, has been used to non-destructively evaluate shrimp freshness by quantifying total volatile bases (TVB), protein content, and chitin, achieving an accuracy of 90.28% on the validation set [261].

Further advancements include the application of nanoporous colorimetric sensor arrays for trimethylamine (TMA) detection in meat, which serves as a key indicator of spoilage [277]. Another study developed a sensor array incorporating nanospheres doped with colorimetric-sensitive materials to assess oyster freshness, achieving high predictive performance with a correlation coefficient of 0.9628 [278].

A silk fibroin fiber (SFF)-based gas sensor has been developed for real-time classification of pork freshness via the detection of total volatile basic nitrogen (TVB-N), exhibiting high reliability and operational flexibility in various conditions [279]. Fluorescence sensing, known for its high sensitivity and rapid response, can also be integrated with IoT frameworks to enable the non-destructive assessment of food chemical composition [155]. Yuan et al. [280] designed a room-temperature multi-gas sensing system capable of accurately quantifying concentrations of ammonia, hydrogen sulfide, and moisture released during pork spoilage. When connected to IoT devices, the system enables real-time evaluation of pork freshness and remote monitoring of its storage and transport conditions, effectively reducing product loss.

In addition, a sensor array based on multiple freshness indicators (e.g., TVB-N, moisture content) combined with AI algorithms has been proposed for non-destructive classification of meat types including buffalo, lamb, and beef [281]. Kim et al. [282] utilized 3D printing technology in conjunction with IoT to develop silk microneedle food sensors capable of penetrating food packaging, collecting spoilage-related compounds via capillary action, and transferring them to integrated detection units. These sensors provide visual indicators of freshness and are particularly suitable for use in retail environments and households.

A novel nanofiber-based time–temperature indicator (TTI) sensor has also been developed for cold chain logistics monitoring [283]. This sensor, embedded within food packaging, transmits real-time data via IoT systems and visually displays temperature fluctuations, thereby enhancing traceability and user accessibility. Furthermore, a hydrogen sulfide (H_2_S) colorimetric sensor utilizing gel-coated silver nanoparticles was created to non-destructively detect H_2_S levels, presenting a visible yellow-to-white color shift for spoilage monitoring in meat products [284]. Zhang et al. [285] reported that low-density polyethylene (LDPE) film incorporating curcumin provides a distinct colorimetric response to beef spoilage at 4 °C, effectively reflecting changes in TVB-N content. Owing to its non-toxic and low-cost properties, this material shows strong potential for large-scale industrial applications in intelligent packaging.

#### 3.3.3. Multimodal Data Fusion Platform Implementation

To enhance the accuracy and efficiency of freshness detection, researchers have increasingly focused on multimodal data fusion techniques, which involve integrating multiple sensing methods to provide a more comprehensive assessment of meat freshness [286]. For instance, one study [287] incorporated spectroscopic technologies such as fluorescence and Raman spectroscopy into an intelligent sensor system capable of online detection of key indicators, including protein content and moisture activity. This system facilitates real-time data sharing via the IoT, thereby supporting remote traceability and compositional analysis in meat processing.

A bionic vision–olfaction co-sensor has been developed to quickly and accurately assess food freshness, while also serving as a human–machine interface for non-destructive inspection [288]. Another approach [289] involves uploading spectroscopic data to the cloud via IoT devices and employing AI and big data analytics for quality assessment, including attributes such as freshness and color.

The integration of electronic nose technology with fluorescence hyperspectral imaging has been shown to significantly improve the prediction accuracy of pork freshness when compared to single-modality systems [290]. Additionally, non-destructive methods using HSI and artificial olfactory systems based on colorimetric sensor arrays have demonstrated success in detecting total viable counts (TVC) to evaluate spoilage in pork products [291]. By fusing spectral and image data from HSI, researchers have been able to rapidly predict lipid oxidation metrics—such as thiobarbituric acid reactive substances (TBARS) and peroxide values (PV)—under varying processing conditions [292].

In practical applications, radio frequency identification (RFID) tags are employed to log the processing history of each meat product. When combined with spectroscopic analysis, these data points enable traceability and verification throughout the supply chain.

Furthermore, a self-calibrating sensor based on amine-responsive fluorescence excitation has been developed for real-time freshness monitoring. This system uses a fluorescent label affixed to filter paper, which can be scanned with a smartphone to provide fast, non-destructive, and visual freshness assessment in products such as pork and shellfish [293]. Another study [294] explored the integration of nano- and biosensors into food packaging or labels to continuously monitor the authenticity, composition, and freshness of various food products—including meats, spices, and seafood—in real time. The combined use of IoT and spectroscopic technologies allows inspection data to be transmitted instantly, empowering consumers to access key food information via smartphones and thereby enhancing transparency and trust.

#### 3.3.4. AI-Driven Freshness Prediction Model Application

AI, particularly ML and deep learning models, has demonstrated significant potential in the real-time, non-destructive prediction of meat freshness. For instance, one study employed an electronic nose in combination with a neural network to monitor beef spoilage under varying storage temperatures. The resulting cluster-fuzzy wavelet neural network (CFWNN) model successfully classified freshness levels and predicted microbial counts, achieving an accuracy of 95.71% for aerobic packaging and 92.95% for modified atmosphere packaging [295].

Another study integrated olfactory imaging with IoT infrastructure by constructing a sensor array and algorithmic framework capable of real-time pork freshness detection. This system incorporates image acquisition, data processing, wireless transmission, and cloud-based analysis, with the trained model achieving an accuracy of 99.8% on the test set [296,297]. These results underscore the feasibility of portable, intelligent detection systems for dynamic freshness monitoring.

Further advancements involve the application of HSI combined with artificial neural networks to quantify TVB-N in chicken meat. The combination of HSI and ant colony optimization (ACO) algorithms enabled rapid, non-invasive estimation of TVB-N levels, offering a promising method for freshness assessment [298]. Additionally, an AI-driven approach integrating Fourier-transform near-infrared (FT-NIR) spectroscopy with fuzzy clustering achieved 93.18% accuracy in classifying pork freshness over varying storage durations, demonstrating the method’s potential for intelligent, non-destructive quality monitoring [299].

Deep learning models, particularly CNNs, have also been applied to detect biogenic amines in meat, achieving a prediction accuracy of 99.29% across various analytes. This approach offers a rapid, cost-effective alternative for real-time freshness monitoring [300]. Kim [301] further developed an AIoT-based meat quality monitoring system, which integrates gas sensors and cameras with a deep learning model to analyze freshness-related data in real time. The system achieved an overall detection accuracy of 99.44% and supports continuous monitoring via an online platform. Furthermore, CNN models were successfully deployed to classify the freshness of beef and lamb in real time, with sensor-triggered LED feedback providing intuitive, high-accuracy freshness indication (99%).

### 3.4. Application in Personalized Nutritional Solution Development

Personalized nutrition refers to the development of customized food products tailored to individual health requirements, dietary preferences, and lifestyle habits. These products not only offer distinctive taste profiles but also ensure a balanced nutritional composition. In the context of meat production, technologies such as cell cultivation and 3D printing have emerged as transformative tools for achieving product personalization.

Cell culture technology enables precise regulation of the muscle-to-fat cell ratio, facilitating the production of meat products with targeted textural and flavor characteristics that meet specific consumer preferences [218,302]. This approach offers high flexibility in tailoring meat composition. In parallel, 3D printing provides a powerful means to customize the nutritional profile of meat by accurately controlling the proportions of macronutrients—such as proteins, lipids, and vitamins—and incorporating functional additives, including antioxidants and prebiotics [303].

Furthermore, 3D printing can address the unique nutritional demands of different demographic groups [304]. For example, meat products with elevated protein content can be formulated for athletes seeking muscle gain and fat loss. For older adults with slower metabolic rates, micronutrient composition can be optimized to support metabolic function. Additionally, individuals with chronic diseases can benefit from meat products designed with medically guided nutritional formulations. Precise control over printing parameters also enables the customization of food texture and mouthfeel to align with consumer sensory expectations [303].

The integration of digital twin factory (DTF) systems further enhances the personalization of meat products. These systems utilize real-time data analytics and ML algorithms to optimize cell growth conditions and simulate potential production issues through virtual modeling. Such predictive modeling not only improves the efficiency and intelligence of the production process but also reduces error rates and resource waste [305,306].

For instance, Zhang proposed a data-driven intelligent customization framework in which consumers can modify product parameters—such as fat-to-lean ratio and marination flavor—in real time via an augmented reality (AR) interface. The system subsequently generates corresponding nutritional analyses and processing solutions, significantly shortening new product development cycles [307]. Similarly, Park et al. introduced a digital twin-driven cyber-physical production system (CPPS), which translates consumer preferences into specific production parameters (e.g., slicing dimensions, seasoning profiles) and uses virtual simulation for feasibility validation, reducing the lead time for customized product delivery by over 30% [308]. Moreover, the health-focused digital twin model developed by Katsoulakis integrates individual metabolic data to design personalized nutrient formulations, such as low-sodium options for hypertensive patients. This approach has progressed to clinical trial stages, demonstrating its practical applicability [309].

While the prospects for personalized nutrition are exciting, moving from proof of concept to large-scale commercial application still requires overcoming a series of significant technical, regulatory, and market challenges. Currently, empirical data on implementation outcomes remain scarce, and the following constraints are particularly prominent: the complexity of nutritional analysis and barriers to clinical integration, the contradiction between high costs and large-scale production, consumer acceptance and concerns about data privacy, and the lag in legal regulations.

Achieving true personalized nutrition is not as simple as mixing a few macronutrients; it requires a deep understanding of nutrient bioavailability, interactions, and the impact of processing on activity. This necessitates high-precision real-time sensing technology and algorithmic models, as well as the establishment of multidisciplinary professional teams to ensure the science and safety of personalization. Additionally, the initial investment and operational costs of 3D printing and cell culture are significantly higher than those of traditional meat production. Customized production processes may struggle to maintain profitability in a broader market context, potentially limiting them to niche, high-end markets rather than becoming mainstream consumer products. Furthermore, consumer acceptance of such foods remains uncertain. Differences in regional, cultural, or lifestyle preferences may lead to consumer skepticism toward “cultured meat,” as people may prefer “natural and traditional” foods. More importantly, personalization may raise issues related to personal privacy and safety. Furthermore, existing food labeling regulations are designed for standardized, mass-produced products. How can compliant, accurate, and easily understandable dynamic nutritional labels be provided for personalized customized products? Regulatory agencies need to establish new approval pathways and labeling standards for this purpose, which is a long and arduous process. Delays in this process may hinder market promotion.

## 4. Techno-Economic Assessment and Outlook

In the wake of AI and the Fourth Industrial Revolution, the meat industry is undergoing a profound transformation toward intelligence and digitalization. However, to turn this vision into reality, it is necessary to go beyond generalities and conduct rigorous quantitative assessments and strategic planning. First, economic feasibility and capital investment are fundamental prerequisites for technology implementation. The application of new technologies, such as intelligent cutting, can indeed increase production by 20–30% and reduce reliance on manual labor by more than half. However, the high investment costs (a complete HPP or intelligent slaughtering system can cost millions of dollars) create a significant barrier to entry. According to industry benchmarks, the return on investment (ROI) typically ranges from 3 to 7 years, and subsequent maintenance costs should not be overlooked. This economic reality has led to a clear disparity in scale: Large enterprises can leverage economies of scale to absorb initial investments and achieve long-term returns, while small and medium-sized enterprises tend to adopt phased, low-cost modular solutions, such as IoT cold chain monitoring, to achieve incremental upgrades. Therefore, the promotion of technology is not uniform but evolves in layers driven by capital.

Similarly, arguments regarding sustainability must be supported by data rather than vague environmental slogans. Life cycle assessment (LCA) studies provide critical quantitative tools for this purpose. For example, PEF technology, a non-thermal sterilization method, can reduce energy consumption by over 50% when combined with other technologies in specific applications. Smart cutting technology increases the yield of bone-in or high-fat meats, directly reducing food waste and, thereby, lowering carbon footprint and water consumption. Additionally, sustainability is reflected in nutritional aspects. Non-thermal processing methods like HPP, due to their low-temperature characteristics, can maximize the retention of heat-sensitive vitamins (such as vitamin B1) in meat products, in stark contrast to the nutrient loss that may occur with traditional thermal processing, directly impacting consumers’ long-term dietary health.

Looking ahead, the meat industry’s industrial upgrading requires a development roadmap based on technology readiness level (TRL) rather than a vague vision. Near-term development should focus on mature technologies at TRL 7–9, such as IoT traceability systems and AI-driven quality visual inspection. The key to promotion lies in reducing barriers to adoption for SMEs through policy incentives (such as tax credits) while simultaneously conducting workforce reskilling. The core challenge in the medium-to-long term lies in overcoming the regulatory challenges associated with potential technologies at TRL 4–6 (such as cell-cultured meat and efficient non-thermal combination technologies).

It is particularly worth emphasizing that AI, non-thermal technologies, and food regulations are forming a profoundly synergistic and mutually shaping relationship. AI plays the role of an ‘intelligent brain,’ empowering the precise application of non-thermal technologies. For example, by analyzing real-time sensor data through ML algorithms, the pressure curve of HPP or the pulse parameters of pulsed electric fields can be dynamically optimized to find the optimal balance between maximizing sterilization effectiveness and minimizing quality damage (such as flavor and texture). However, this dynamic, adaptive processing model combining AI and non-thermal technology poses significant challenges to the current traditional food regulatory framework, as it raises the regulatory dilemma of ‘how to validate a “black box” decision-making process.’ As a result, future food regulations must evolve toward a ‘data-driven’ and ‘results-oriented’ model. This means that regulatory authorities must shift their focus from approving fixed process parameters to evaluating the performance of AI systems and verifying their continuous, traceable outcomes. Therefore, innovative mechanisms such as ‘regulatory sandboxes’ will become crucial, as they allow new technologies to be tested in controlled environments, providing valuable data and practical experience for developing new regulations adapted to intelligent production.

In summary, the future transformation of the meat industry is a complex systemic engineering project. Only by combining technological innovation with rigorous economic analysis, quantitative sustainability assessments, and structured development pathways, through collaborative efforts between government, research institutions, and the industry, can the meat industry truly seize the historical opportunities of Industry 4.0. At that point, the industry will no longer be merely a production sector meeting consumer demand but will advance to new heights of social responsibility and environmental management, ultimately achieving the deep integration and mutual benefit of economic and social benefits.

## 5. Conclusions

The meat industry is undergoing a fundamental transformation driven by Industry 4.0 and AI. This review examined key innovations—from non-thermal physical technologies (PEF, ultrasound, HPP) to digital systems (IoT, blockchain, AI), and emerging approaches like cultured meat and 3D bioprinting—that are reshaping processing efficiency, quality, and traceability. These technologies offer clear advantages in enhancing product safety, optimizing resource use, and enabling personalized nutrition. However, challenges such as high capital investment, lack of standardization, limited scalability, and regulatory uncertainty continue to constrain widespread adoption. Sustainability metrics like lifecycle emissions, water usage, and long-term stability also require deeper validation. Moving forward, efforts should focus on cross-disciplinary integration, unified technical standards, cost-effective deployment strategies, and greater public engagement. Advancing these fronts will be critical to realizing a truly intelligent, resilient, and sustainable meat production system.

## Data Availability

No new data were created or analyzed in this study. Data sharing is not applicable to this article.
